# Binding of the Anti-FIV Peptide C8 to Differently Charged Membrane Models: From First Docking to Membrane Tubulation

**DOI:** 10.3389/fchem.2020.00493

**Published:** 2020-06-26

**Authors:** Daniele Di Marino, Agostino Bruno, Manuela Grimaldi, Mario Scrima, Ilaria Stillitano, Giuseppina Amodio, Grazia Della Sala, Alice Romagnoli, Augusta De Santis, Ornella Moltedo, Paolo Remondelli, Giovanni Boccia, Gerardino D'Errico, Anna Maria D'Ursi, Vittorio Limongelli

**Affiliations:** ^1^Department of Life and Environmental Sciences, New York-Marche Structural Biology Center (NY-MaSBiC), Polytechnic University of Marche, Ancona, Italy; ^2^Department of Pharmacy, University of Naples “Federico II”, Naples, Italy; ^3^Department of Pharmacy, University of Salerno, Fisciano, Italy; ^4^Department of Medicine, Surgery and Dentistry “Scuola Medica Salernitana”, University of Salerno, Baronissi, Italy; ^5^Department of Neuroscience, Psychology, Drug Research and Child Health, University of Florence, Florence, Italy; ^6^Department of Chemical Science, University of Naples Federico II, Naples, Italy; ^7^Faculty of Biomedical Sciences, Institute of Computational Science, Università della Svizzera italiana (USI), Lugano, Switzerland

**Keywords:** NMR, HIV, membrane tubulation, MPER, FIV, peptide/membrane binding, molecular dynamics, umbrella sampling

## Abstract

Gp36 is the virus envelope glycoproteins catalyzing the fusion of the feline immunodeficiency virus with the host cells. The peptide C8 is a tryptophan-rich peptide corresponding to the fragment ^770^W-I^777^ of gp36 exerting antiviral activity by binding the membrane cell and inhibiting the virus entry. Several factors, including the membrane surface charge, regulate the binding of C8 to the lipid membrane. Based on the evidence that imperceptible variation of membrane charge may induce a dramatic effect in several critical biological events, in the present work we investigate the effect induced by systematic variation of charge in phospholipid bilayers on the aptitude of C8 to interact with lipid membranes, the tendency of C8 to assume specific conformational states and the re-organization of the lipid bilayer upon the interaction with C8. Accordingly, employing a bottom-up multiscale protocol, including CD, NMR, ESR spectroscopy, atomistic molecular dynamics simulations, and confocal microscopy, we studied C8 in six membrane models composed of different ratios of zwitterionic/negatively charged phospholipids. Our data show that charge content modulates C8-membrane binding with significant effects on the peptide conformations. C8 in micelle solution or in SUV formed by DPC or DOPC zwitterionic phospholipids assumes regular β-turn structures that are progressively destabilized as the concentration of negatively charged SDS or DOPG phospholipids exceed 40%. Interaction of C8 with zwitterionic membrane surface is mediated by Trp1 and Trp4 that are deepened in the membrane, forming H-bonds and cation-π interactions with the DOPC polar heads. Additional stabilizing salt bridge interactions involve Glu2 and Asp3. MD and ESR data show that the C8-membrane affinity increases as the concentration of zwitterionic phospholipid increases. In the lipid membrane characterized by an excess of zwitterionic phospholipids, C8 is adsorbed at the membrane interface, inducing a stiffening of the outer region of the DOPC bilayer. However, the bound of C8 significantly perturbs the whole organization of lipid bilayer resulting in membrane remodeling. These events, measurable as a variation of the bilayer thickness, are the onset mechanism of the membrane fusion and vesicle tubulation observed in confocal microscopy by imaging zwitterionic MLVs in the presence of C8 peptide.

## Introduction

**Figure 1:**
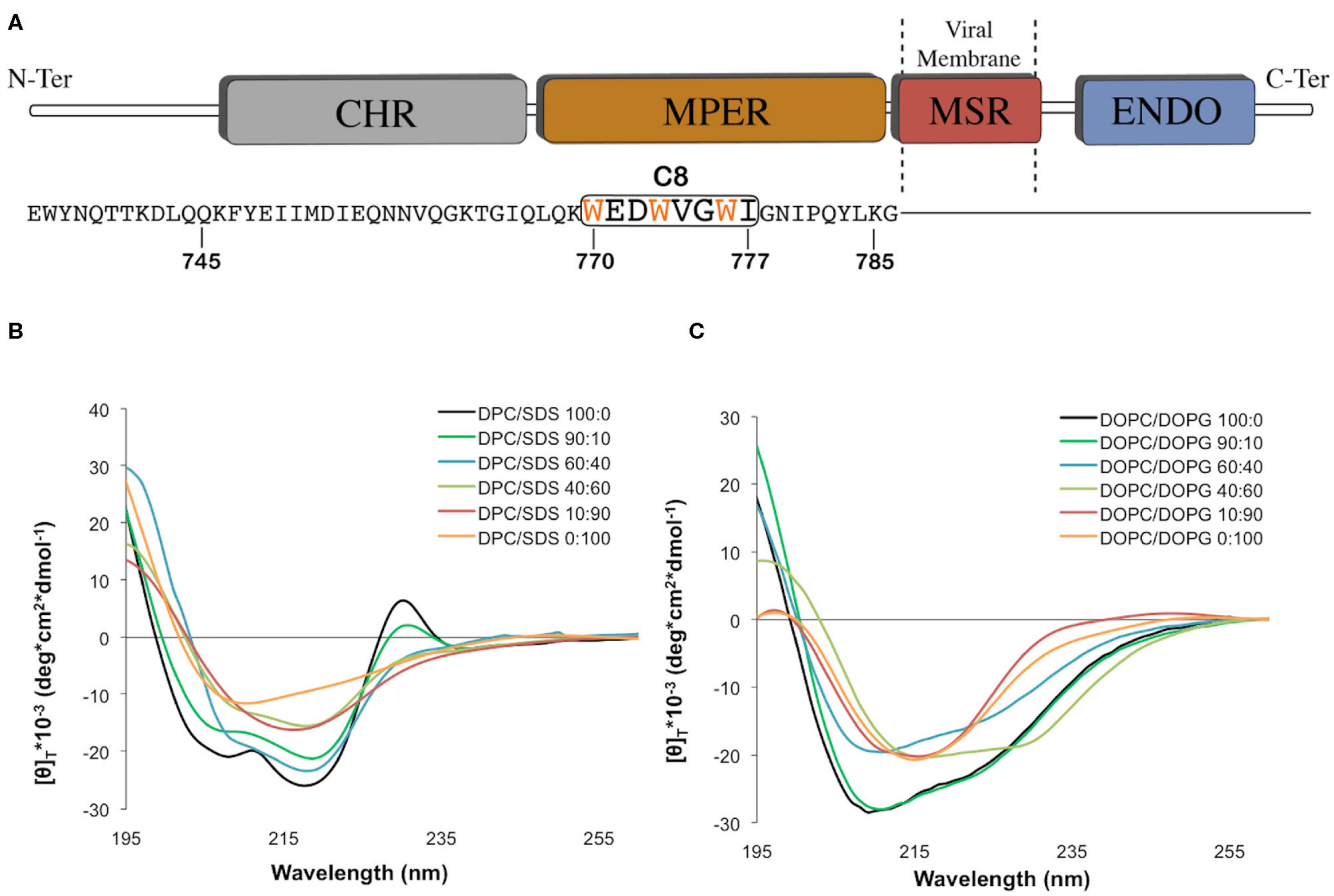
**(A)** Graphic representation of the C-terminal domains of the feline immunodeficiency virus (FIV) coat glycoprotein gp36-CHR. The C-terminal R-helix region (CHR), the Membrane-Proximal External Region (MPER), the Membrane-Spanning Region (MSR), and the interior region (ENDO) are schematically reported with different colors. The C8 peptide sequence is underlined by the black box, and the three tryptophans are reported in orange. **(B)** Normalized circular dichroism (CD) spectra of C8 in different: (90:10, 60:40, 40:60, 10:90) CD spectra in DPC/SDS 100:0 and 0:100 were previously published (Scrima et al., 2014). **(C)** Normalized circular dichroism (CD) spectra of C8 in different DOPC/DOPG SUV at molar ratio: 100:0, 90:10, 60:40, 40:60, 10:90, and 0:100. The CD spectra were acquired using a JASCO J810 spectropolarimeter at room temperature with a cell path length of 1 mm. The measurement range spans from 190 to 260 nm. The C8 spectra in pure DPC and SDS micelle solutions were taken from our previous work (Scrima et al., 2014).

Gp36 and gp41 are virus envelope glycoproteins catalyzing the fusion of FIV and HIV with their respective host cells. Gp36 and gp41 have a common structural framework, including fusion peptide (FP), N-terminal heptad repeat (NHR), C-terminal heptad repeat (CHR), and membrane proximal extracellular region (MPER) (Pancino et al., 1993, 1995; Wyatt and Sodroski, 1998; Eckert et al., 1999; Frey et al., 2001). Virus entry is successful if NHR and CHR fold back to form a low energy stable six-helical bundle (6HB) (Chan et al., 1997; Weissenhorn et al., 1997; Lamb and Jardetzky, 2007; Harrison, 2008), while MPER drives the correct positioning of the glycoprotein on the host cell membrane (Figure 1A; Lorizate et al., 2008; Sun et al., 2008; Liu et al., 2018). The design of molecules interfering with the formation of the six-helical bundle or inhibiting the correct juxtaposition of virus and cell membrane is a strategy currently pursued to design new anti-viral entry inhibitors. Peptides derived from gp41 CHR and NHR have been extensively investigated, and enfuvirtide is an entry inhibitor currently used for anti-HIV therapy, acting by preventing the correct interaction of gp41 NHR with the respective CHR region (Kliger et al., 2001; Dwyer et al., 2007; Steffen and Poehlmann, 2010; Cai et al., 2011). MPER is a hydrophobic, Trp-rich region (Figure 1A) characterized by membrane affinity and conformational plasticity (Muñoz-Barroso et al., 1999; Salzwedel et al., 1999; Buzón et al., 2010). In gp41, this region includes several immunogenic epitopes reason why it is considered an attractive target to develop anti-HIV vaccines (Buzón et al., 2010; Huang et al., 2012); conversely in gp36 MPER immunogenicity has a minor role. Therefore it is rather studied for the development of new anti-FIV entry inhibitors (Lombardi et al., 1996; Massi et al., 1997).

In this context, we previously studied an octapeptide (C8) corresponding to the fragment ^770^W-I^777^ of gp36 MPER (Figure 1A; Lombardi et al., 1996; Giannecchini et al., 2003). C8, including three equally spaced Trp residues, exhibited powerful antiviral activity on all the FIV isolates tested. Investigation of C8 biological activity pointed to a molecular mechanism based on (i) the binding of C8 with the gp36 NHR sequence to prevent the formation of 6HB. C8 activity was blocked by a peptide derived from the N-terminal portion of gp36 (Lorizate et al., 2008; Sun et al., 2008); (ii) the interaction of C8 with the lipid bilayers, to inhibit the gp36-MPER/membrane anchoring. Several experimental evidence has proved that Trp, residues that have a critical role in stabilizing the peptide/ lipid membrane interaction, are essential for the biological action of C8 (D'Ursi et al., 2003, 2006; Grimaldi et al., 2018, 2020).

Increasing evidence shows that during the fusion of the virus with the host cell membrane, translational diffusion of the lipids induces a change in the bilayer composition (Chernomordik and Kozlov, 2008). Lipid composition affects the structure and function of envelope glycoprotein, particularly of those regions, such as MPER responsible for interaction with lipid membranes. On these bases, using several different biophysical techniques, we previously studied C8 in lipid vesicles of different compositions, including saturated and unsaturated phospholipids. In particular, as the lipid composition of viral envelopes is usually rich in sphingolipids and cholesterol (CHOL), we studied the effect of C8 on membranes, including POPC/sphingomyelin (SM)/CHOL in different ratios (D'Ursi et al., 2003, 2006; Giannecchini et al., 2003, 2005, 2007; Merlino et al., 2012; Scrima et al., 2014).

The charge of the plasma membrane is critical to regulating a plethora of vital biological functions. As an exemplum, a modest variation of membrane charge proved to be the cause of the onset of antibiotic resistance against antimicrobial peptides or determined dramatic loss of biological activity in peptides and proteins involved in cell membrane remodeling (Esposito et al., 2006; D'Errico et al., 2009; Merlino et al., 2012; Scrima et al., 2014; Kelley et al., 2015; Oliva et al., 2015; Khondker et al., 2019). Prompted by these data, we studied the structure of C8 in membrane mimicking systems characterized by different surface charges. In particular, we chose micelle solutions, including negatively charged sodium dodecyl sullphate (SDS) and zwitterionic dodecylphosphocholine (DPC) (Scrima et al., 2014). In the present work, we extend our study on the effect exerted by the membrane charge on C8, by analyzing its structural behavior on differently charged lipid membranes, each containing a specific ratio of zwitterionic and negatively charged phospholipids. All the systems have been investigated through a bottom-up multiscale protocol, including circular dichroism (CD), nuclear magnetic resonance (NMR), electron spin resonance (ESR) spectroscopy, atomistic molecular dynamics simulations, and confocal microscopy. In addition to the conformational data derived from the standard 2D NMR experiments in SDS/DPC mixed micelle solution, ESR, confocal microscopy imaging, and funnel molecular dynamic calculation, make it possible to take attention on the effect exerted by C8 on multilamellar vesicles (MLVs). Funnel molecular dynamics (FMD) calculations, supported by spectroscopic data, allowed rationalizing structural events that confocal microscopy permits to observe in images.

Interestingly, depending on negative charged/zwitterionic phospholipid proportion, C8 induces a variation of vesicle size and shape, generating a dense network of membrane tubes. This effect, typical of membrane remodeling processes, and unusual for short peptides like C8, suggests a possible development of C8 and its new derivatives in drug discovery as antiviral, and also in biomedicine, as a tool to favor membrane trafficking and cell communication. Finally, our results endorse the use of multiscale approaches to elucidate key structural questions related to the conformational plasticity and the chameleon-like activity of the MPER region.

## Materials and Methods

### Peptide Synthesis

C8 peptide was synthesized according to the procedure published elsewhere (Scrima et al., 2014). Nitrobenzodiazole (NBD) labeled C8 peptide was prepared by synthesizing Ac-Dap(NBD)-Glu-Asp-Trp-Val-Gly-Trp-Ile-CO-NH_2_ including Dap (L-diamino propionic acid) amino acid in substitution of N-terminal Trp. Fmoc solid-phase synthesis method was employed using Dap(NBD)-OH reagent. This was obtained by alkylating the free amino group of Nα-Fmoc, L-diamino propionic acid (Fmoc-Dap-OH) with NBD chloride (Dufau and Mazarguil, 2000). Dichloromethane and methanol, HPLC-grade solvents, were obtained from Sigma-Aldrich (St. Louis, MO).

### MLVs Preparation

The six MLVs systems of (1,2-dioleoyl-sn-glycero-3-phosphocholine) DOPC/[1,2-dioleoyl-sn-glycero-3-phospho-(1′-rac-glycerol)] DOPG (Figure S1) with different molar ratio (i.e., 100:0, 90:10, 60:40, 40:60, 10:90, and 0:100) were prepared mixing appropriate amounts of lipids, dissolved in dichloromethane/methanol mixtures (2:1 v/v, 10 mg/mL lipid concentration), in a round-bottom test tube. The total weight of the lipid for each sample was 0.3 mg. MLVs were prepared from lipid DOPC and DOPG solution, which was desiccated, dried overnight and hydrated in a phosphate buffer at pH 6.8. MLVs in the presence of labeled-C8 peptide were prepared by adding labeled-C8 peptide at a concentration of 40 μM (lipid to peptide molar ratio 50:1). The solution of lipids in the absence or presence of labeled C8 peptide was used for confocal microscope analysis. For the preparation of the SUVs, MLV containing C8 peptide was sonicated for 30 min. The phospholipids DOPC, DOPG were purchased from Avanti Polar Lipids (Birmingham, AL, USA).

### Confocal Microscope Imaging

Images were acquired as previously described (Fasano et al., 2018; Amodio et al., 2019) on a laser scanning confocal microscope (LSM 510; Carl Zeiss MicroImaging) equipped with a Plan Apo 63X, NA 1.4 oil immersion objective lens. Ten microliter of MLV solution, in the presence or absence of NBD labeled-C8 peptide, was taken and spotted on a coverslip. For each field, both fluorescent and transmitted light images were acquired, at room temperature, on separate photomultipliers and analyzed with Zeiss LSM 510 4.0 SP2 software. In samples in which different z-planes were distinguishable, a z-stack acquisition mode was performed to focus on a single z-plane as published elsewhere (Schneider et al., 2012).

### Circular Dichroism Spectroscopy

Circular Dichroism (CD) experiments were performed at room temperature on an 810-Jasco spectropolarimeter, using a quartz cuvette with a path length of 1 mm. CD spectra were acquired at 25 °C using a measurement range from 190 to 260 nm, a bandwidth of 1 nm, four accumulations, and a scanning speed of 10 min. During all the measurements, the trace of the high tension voltage was verified to be <700 V, which should ensure the reliability of the data obtained. Spectra, corrected for the solvent contribution, were acquired on samples containing the peptide (1 × 10^−4^ M) in DPC/SDS (Figure S1) molar ratio (90:10, 60:40, 40:60, and 10:90) solved in 10 mM phosphate buffer at pH 6.8. We also acquired CD spectra of C8 in a solution containing small unilamellar vesicles (SUVs). These included different DOPC/DOPG (i.e., 100:0, 90:10, 60:40, 40:60, 10:90, and 0:100) molar ratios. Estimation of secondary structure content was carried out using the algorithms CONTIN and SELCON from the DICHROWEB website (Whitmore and Wallace, 2004).

### NMR Analysis

NMR spectra were recorded on a Bruker DRX-600 spectrometer at 300 K. The dry peptide was dissolved in DPC/SDS micelles solution with different molar ratios (i.e., 90:10, 60:40, 40:60, and 10:90 in 10 mM phosphate buffer (H_2_O/D_2_O) at pH 6.8). 1D ^1^H homonuclear spectra were recorded in the Fourier mode, with quadrature detection. Two-dimensional (2D) spectra of C8 in the final micelle solutions were recorded. 2D ^1^H homonuclear TOCSY and NOESY were run in the phase-sensitive mode using quadrature detection in ω1 by time-proportional phase incrementation of the initial pulse (Jeener et al., 1979; Piantini et al., 1982; Bax and Davis, 1985). The water signal was suppressed using WATERGATE pulse sequence experiments (Piotto et al., 1992). Data block sizes were 2,048 addresses in t2 and 512 equidistant t1 values. Before Fourier transformation, the time domain data matrices were multiplied by shifted sin^2^ functions in both dimensions. A mixing time of 70 ms was used for the TOCSY experiments. NOESY experiments were run at 300 K with mixing times of 250 ms. Qualitative and quantitative analyses of DQF-COZY, TOCSY, and NOESY spectra were achieved using SPARKY software (Goddard and Kneller, 2004).

### NMR Structure Calculations

Peak volumes were translated into upper distance bounds with the CALIBA routine from the CYANA software package (Guntert et al., 1997). The requisite pseudo atom corrections were applied for non-stereo specifically assigned protons at prochiral centers and the methyl group. After discarding redundant and duplicated constraints, the final list of experimental constraints was used to generate an ensemble of 100 structures by the standard CYANA protocol of simulated annealing in torsion angle space implemented (using 10,000 steps). No dihedral angle or hydrogen bond restraints were applied. The best 50 structures that had low target function values and small residual violations were refined by in vacuo minimization in the AMBER 1991 force field using the SANDER program of the AMBER 5.0 suite (Pearlman et al., 1995). To mimic the effect of solvent screening, all net charges were reduced to 20% of their real values. A distance-dependent dielectric constant (ε = r) was used. The cut-off for unbounded interactions was 12 Å. NMR-derived upper bounds were imposed as semi-parabolic penalty functions, with force constants of 16 Kcal/mol Å^2^. The function was shifted to be linear when the violation exceeded 0.5 Å. The best ten structures after minimization had AMBER energies ranging from −441.4 to −391.1 Kcal/mol. Final structures were analyzed using the Insight 98.0 program (Molecular Simulations, San Diego, CA, USA).

### ESR Spectroscopy

Spin-labeled phosphatidylcholine (n-PCSL) was added to the lipid mixture (1% wt %/wt on the total lipid) by mixing appropriate amounts of a spin-label solution in ethanol (1 mg/mL) with the lipid organic mixture. A thin lipid film was produced by evaporating the solvents with dry nitrogen gas, and final traces of solvents were removed by subjecting the sample to vacuum desiccation for at least three h. The samples were then hydrated with 30 μL of 10 mM phosphate buffer at pH 6.8 and repeatedly vortexed, obtaining a MLV suspension. This suspension was transferred into a 25 μL glass capillary and immediately sealed. Samples containing the peptide were prepared similarly, except that the lipid film was hydrated directly with the peptide solution phosphate buffer at pH 6.8 (peptide concentration of 2 mg/mL). Electron spin resonance spectra of lipid and lipid/peptide samples were recorded on a 9-GHz Bruker Elexys E-500 spectrometer (Bruker, Rheinstetten, Germany). Capillaries containing the samples were placed in a standard 4-mm quartz sample tube, and the temperature was maintained constant during the measurement. The instrumental settings were as follows: sweep width 120 G, resolution 1.024 points, modulation frequency 100 kHz, modulation amplitude 1.0 G, the time constant 20.5 ms, incident power 5.0 mW. Several scans, typically 16, were accumulated to improve the signal-to-noise ratio. The values of the outer hyperfine splitting, 2A_max_, were determined by measuring the difference between the low-field maximum and the high-field minimum (Schorn and Marsh, 1997; Marsh and Horvath, 1998). The primary source of error on the 2A_max_ value is the uncertainty in the composition of samples prepared by mixing a few microliters of mother solutions. For this reason, the reproducibility of 2A_max_ determination was estimated by evaluating its value for selected independently prepared samples with the same nominal composition. It was found to be § 0.2–0.3 G. The spin-labeled n-PCSL, with the nitroxide group at different positions, n, in the sn-2 acyl chain were purchased from Avanti Polar Lipids (Birmingham, AL, USA). The spin-labels were stored at −20°C in ethanol solutions at a concentration of 1 mg/mL.

### Computational Protocol

#### System Parameterization

The C8 starting structure was obtained from the NMR experiments described above and then parameterized using the ff99SBildn, force fields of Amber14 suite (http://ambermd.org). Six bilayers with different DOPC/DOPG molar ratio using charmm-gui.org (http://charmm-gui.org) and parameterized with lipid11 force field (http://ambermd.org) were built: (i) pure DOPC, (ii) DOPC/DOPG 90:10, (iii) 60:40, (iv) 40:60, (v) 10:90, and (vi) pure DOPG. The absolute number of the lipids molecule in each system is reported in the Table S1.

#### Systems Setup and MD Simulations

All the simulations were carried out using the NAMD 2.9 software (Kalé et al., 1999; http://www.ks.uiuc.edu/Research/namd/) and TIP3P as water solvation model. Each C8-bilayer system was solvated using the VMD autoionize plugin (Humphrey et al., 1996; http://www.ks.uiuc.edu/Research/vmd/). The box dimension was equal to 70 × 70 × 155 Å, with a total amount of 91,470, 90,764, 91,404, 91,014, 92,904, and 94,865 atoms for C8 in pure DOPC, DOPC/DOPG 90:10, 60:40, 40:60, 10:90, and pure DOPG, respectively. Each system was equilibrated as follows: (i) 4,000 steps of conjugate gradient minimization, where harmonic constraints on the system were progressively reduced; (ii) 90 ps of heating phase from 50 to 300 K using the Langevin thermostat, where harmonic constraints on the lipid atoms were progressively reduced; (iii) 70 ps of pressure equilibration with a target pressure of 1.01325 bar and using the Nosè-Hoover Langevin barostat, where harmonic constraints were gradually switched off; (iv) 100 ps of pressure equilibration without constraints; (v) 50 ns of preparatory phase; and (vi) 100 ns of production phase. The production phase was carried out using the periodic boundary conditions in the NPT ensemble. The time-step was set to 2 fs, and the RATTLE algorithm was used for the hydrogen atoms. The temperature was retained at 300 K using the Langevin thermostat. A cut off of 12 Å was set for both electrostatic and van der Waals short-range interactions, while the long-range electrostatics were treated using the PME methodology (Darden et al., 1993; Anzini et al., 2008, 2011) with a grid spacing of 1.0 Å and an interpolation order of 4. The sum of preparatory and production runs generated 150 ns for each of the six different systems [i.e., (1) pure DOPC, (2) DOPC/DOPG 90:10, (3) 60:40, (4) 40:60, (5) 10:90, and (6) pure DOPG] for a total of 0.9 μs. All the analyses were performed only on the 100 ns of the production runs of each simulation since the first 50 ns (i.e., preparatory phase) were performed to equilibrate the lipids bilayer.

#### Generation of the Peptide-Membrane Complexes and FMD Simulations

The initial distance between the center of mass of the peptide and the center of mass of the membrane was set to 35 Å. The obtained systems were submitted to a relaxation protocol as described in the System Setup and MD simulations section. A preparatory phase was conducted for 50 ns in the NPT ensemble with a target pressure equal to 1.01325 bar, a time-step of 2 fs, and using the RATTLE algorithm for the hydrogen atoms. The temperature was retained at 300 K using the Langevin thermostat. During the 50 ns preparatory phase, the position of the peptide relative to the membrane was restrained to keep C8 in the unbound state. The distance between the closest pair of atoms belonging to the peptide and the membrane was constrained to values higher than 15 Å. For each C8-membrane system, the last frames of the 50 ns preparatory phase were used as starting structures to run an independent run of FMD simulations (i.e., production phase; 100 ns long, each one; Limongelli et al., 2013). The conformational space to explore was reduced using a funnel-restrained potential. This potential is a combination of a cone restraint, which includes the external side of the membrane, and a cylindrical part, which is directed toward the solvent. We set the alpha angle to 0.8 rad, Rcyl to 1 Å and Zcc to 35 Å from the center of the membrane. Using the funnel potential during the simulation, as the peptide reaches the edge of the funnel, a repulsive bias is applied to the system, disfavouring it from visiting regions outside the funnel. Finally, the frames where the system feels the external potential were not considered for the statistical analysis of the simulations.

#### Analysis of the Distance and the Number of Contacts Between the C8 Peptide and the Membrane

We analyzed the minimum distance (i.e., CV2) and several contacts formed between the C8 peptide and the polar head of both DOPC and DOPG phospholipids (i.e., CV1) during the six different FMD simulations. The analysis was performed using the g_mindist tool of Gromacs V. 4.6.7 (Spoel et al., 2014) with a contacts cut-off of 4 Å. The CV2 was calculated between the Cα atom of Val5 in C8 and the atoms of the polar heads of both DOPC and DOPG phospholipids. We note that the Cα atom of Val5 is situated in the C8 secondary structure and well-represents the motion of the peptide relative to the membrane plane. Therefore, the distance between the Cα of Val5 and the head of the lipids is used as a collective variable to evaluate the peptide-membrane distance and to detect the binding events. Differently, considering the peptide center of mass, hence including the more flexible amino acids at the C-terminus, would make the CV2 values noisy, being dependent not only on the position of the peptide concerning the membrane but also on the conformations assumed by the peptide during the simulation. From this analysis, we have defined two parameters to classify the binding states of the peptide. The C8 peptide is formed by 8 amino acids for a total of 140 atoms, so that several contacts (i.e., CV1) higher than 200 and 250, means that at least 70 and 90% of the peptide atoms, respectively, can establish a minimum of 2 contacts with the atoms of the lipids polar heads. Similarly, a distance (i.e., CV2) lower than 0.5 and 0.7 nm between the Cα atom of Val5 and the center of mass of the polar heads, represents less than half (0.5 nm) and few more than half (0.7 nm), respectively, of the cut off used to calculate electrostatic interactions in MD simulation. The binding states of the peptide were classified as follows: (i) unbound: number of contacts significantly lower than 200 and distance higher than 0.7; (ii) superficial binding: number of contact higher than 200 but lower than 250 and distance lower than 0.7 nm; (iii) strong binding: number of contact higher than 250 and distance lower than 0.5 nm.

#### Cluster Analysis

The C8 peptide-binding conformations reported in **Figure 6** were obtained from a conformational cluster analysis of the FMD simulations. The clustering was carried out using the GROMOS algorithm that is included in g_cluster tool of Gromacs V. 4.6.7 (Spoel et al., 2014). The clustering was performed on the RMSD (Root Mean Square Deviation) value of the Cα atoms of the whole peptide core (1–8 aa) using a cut-off of 2 Å. The representative conformations (i.e., centroids) of the cluster families were extracted based on the two CVs described in the previous paragraph (i.e., number of contacts between the C8 and the polar heads of the phospholipids, and the minimum distance between the Cα atom of the Val5 of the C8 and the atoms of the polar heads of the phospholipids). The size and the distribution relative to the simulation time of each family obtained through the clustering approach in pure DOPC and DOPC/DOPG 90:10 are reported in **Figure 5**. The centroids of the two most populated families showing strong binding modes (according to the previously defined CVs intervals) were selected and compared to the NMR structures of C8 peptide. All the images showing the three-dimensional (3D) structure of the C8 peptide and C8-membrane interaction were obtained using UCSF Chimera v1.10.1 and Pymol v1.7.2.1 software (http://www.pymol.org).

#### Umbrella Sampling (US)

The free energy profile for the translocation of the C8 peptide across the pure DOPC membrane was estimated using the US technique (Kästner, 2011). The peptide was pulled into the membrane to generate the structures for the US. The pulling simulation was performed for 100 ns in which a harmonic potential with a constant force of 1,000 kJ/(mol × nm^2^) was applied between the COM of the C8 peptide and a reference position along the *Z*-axis. This reference position represents the COM of the membrane. The structures from the pulling trajectory were selected every 1.0 Å in a range in which *Z* = 50 Å represents the peptide in the bulk water, and *Z* = 0 Å represents the peptides, buried in the membrane (i.e., into the center of the DOPC bilayer). The procedure above generated 50 windows, and each window was simulated up to 50 ns for a total of 2.5 μs. To calculate the PMF, a harmonic potential with a force constant *k* = 1,000 kJ/(mol × nm^2^) acting on the peptide COM along the z-coordinate (i.e., reaction coordinate) was employed. The data collected in each window were then analyzed using the WHAM algorithm (Kumar et al., 1992).

#### Membrane Thickness Calculation

To quantify the membrane thickness, we computed the local 1-D electron density profile in pure DOPC (i.e., 100:0 DOPC/DOPG) and DOPC/DOPG 90:10 membranes, projected along the bilayer normal. The density profile computations were performed with the VMD plugin Density Profile Tool (https://github.com/giorginolab/vmd_density_profile).

## Results

### The C8 Structure: CD and NMR Spectroscopy

Micelle solutions are largely used as biomimetic membrane models to study the structural features of membranotropic molecules. From the technical point of view, they are ideal systems for CD and NMR analysis in solution, as they tumble sufficiently quickly to result in high-resolution spectral lines (Mannhold et al., 2006; Foster et al., 2007; Pandey et al., 2012). Accordingly, we studied C8 (Figure 1A) by CD and NMR spectroscopy in mixed micelle solutions composed of different proportions of zwitterionic (DPC), and negatively charged (SDS) detergents. Figure 1B shows CD spectra collected on samples containing C8 peptide (5.0 × 10^−4^ M) in DPC/SDS molar ratio (90:10, 60:40, 40:60, and 10:90 M/M). For comparison, we also show the previously published (Scrima et al., 2014) CD spectra in pure DPC and SDS micelles. The concentrations of SDS and DPC were ten-fold higher than the critical micellar concentration (c.m.c.) (DPC 8.1 mM and SDS 1.1 mM). Figure 1B shows that the shapes of the CD spectra vary in dependence of lipid composition. Quantitative analysis of CD curves, using CONTIN algorithm (DICHROWEB), indicates that in micelle solution containing an excess of zwitterionic DPC (i.e., DPC/SDS 100:0, 90:10, and 60:40), C8 assumes prevalent turn-helical structure. In micelle solution containing an excess of negatively charged SDS (i.e., DPC/SDS 40:60, 10:90, and 0:100), a decrease of turn-helical structure is observable, consistent with CD curves typical of equally distributed turn-helical and extended-random coil conformations.

In order to study the effect of membrane charge on C8 conformation by using more biomimetic models, CD experiments were also recorded in DOPC/DOPG SUVs. SUVs included DOPC/DOPG proportions as previously reported (DOPC/DOPG 100:0, 90:10, 60:40, 40:60, 10:90, and 0:100 M/M) (Figure 1C). As observed for the micelle systems, in the conditions characterized by the excess of zwitterionic phospholipids, i.e., SUVs solutions containing 60:40, 90:10, and 100:0 DOPC/DOPG molar ratio, C8 assumes prevalent turn-helical structures. In conditions characterized by the excess of negatively charged phospholipids, i.e., SUVs solutions containing 40:60, 10:90, and 0:100 DOPC/DOPG molar ratio, C8 assumes prevalent extended-random coil conformations. Interestingly, in both the micelle and SUVs membrane mimicking systems, C8 undergoes a conformational transition when the proportion of zwitterionic/negatively charged phospholipid changes from 60:40 to 40:60 molar ratio.

NMR spectra were acquired in the same DPC/SDS mixture used for the CD measurement but using 0.1 mM peptide concentration. To exclude potential aggregation, we recorded the 1D proton spectra at a concentration range spanning 1–0.1 mM. At a peptide concentration of 0.1 mM, the peptides did not display any noticeable effects of aggregation. Chemical shift assignments of the proton spectra were achieved via the standard systematic application of DQF-COZY, TOCSY, and NOESY experiments, using the SPARKY software package (Goddard and Kneller, 2004) according to the procedure of Wüthrich (Jeener et al., 1979; Piantini et al., 1982; Bax and Davis, 1985; Wuthrich, 1986). The values of the proton chemical shifts are reported in Tables S2–S7; a careful inspection of C8 chemical shifts in the different environmental conditions indicates that NH and CHα of Trp1 and Glu2 signals are significantly affected by the changes in micelle composition.

**Figure 2:**
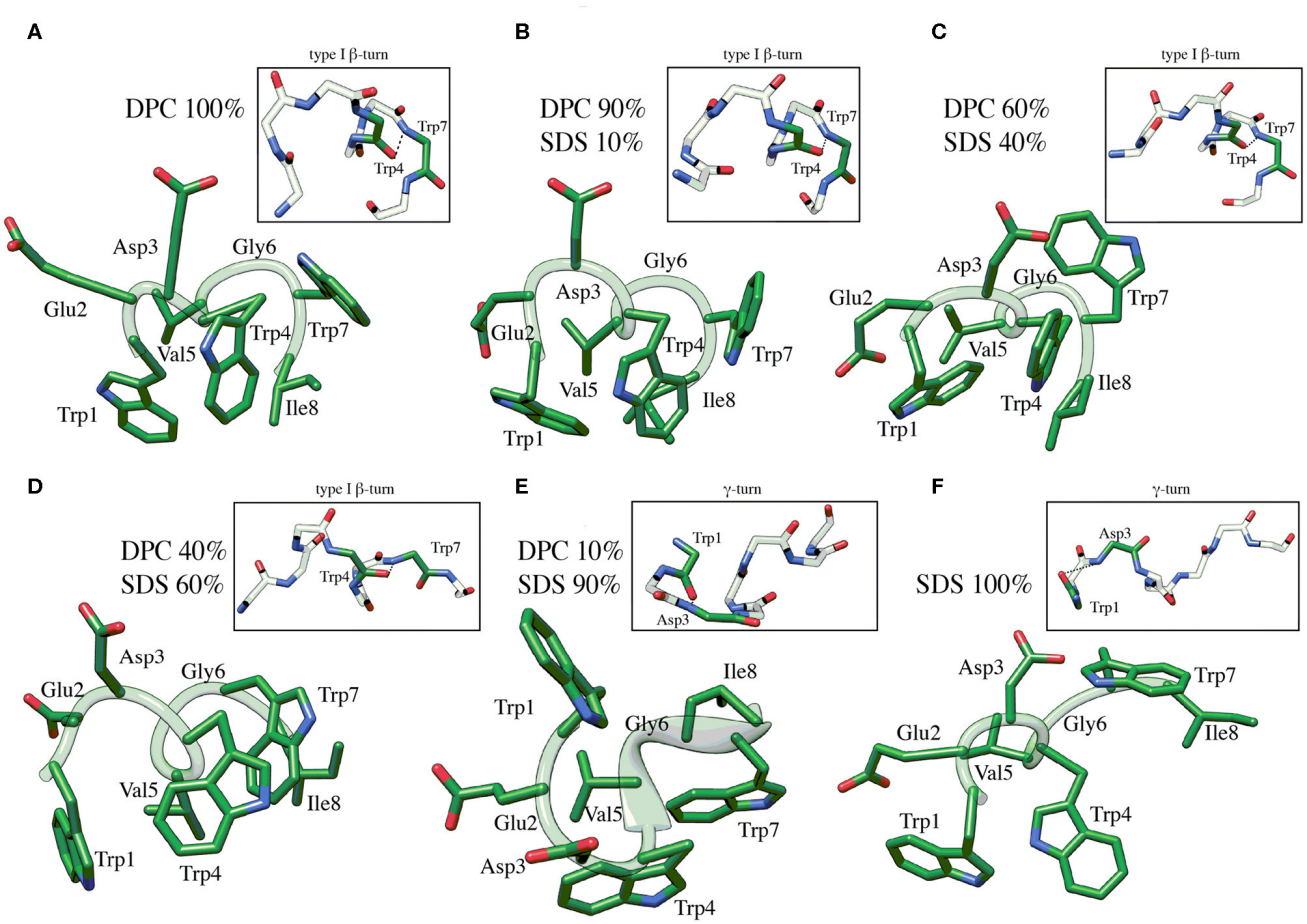
Low energy NMR models calculated for C8 in DPC/SDS molar ratio: 90:10 **(B)**, 60:40 **(C)**, 40:60 **(D)**, 10:90 **(E)**. NMR models in pure DPC **(A)** and in pure SDS **(F)** were previously published (Scrima et al., 2014), and they are now shown for comparison. The calculation was carried out using standard CYANA protocol of simulated annealing in torsion angle space (10,000 steps) applying interprotonic distances as restraints collected from NOE data. The H-bond between the carbonyl O of Trp4 and the amide NH of Trp7 that is specific for the type I β-turn structure is reported in the insets of **(A–D)**. The H-bond between the carbonyl O of Trp1 and the amide NH of Asp3 that is specific for the γ-turn structure is reported in the insets of **(E,F)**.

C8 NMR structures were calculated by simulated annealing procedures based on sequential and medium-range NOE-derived restraints extracted from NOESY spectra (see Figure S2). The best 50 structures were chosen according to the lowest values of the penalty (f) for the target function. These structures were energy minimized using the distance restraints with a progressively smaller force constant. The minimization procedure yielded an improved helical geometry and lower total energy of the structures (Figure 2; Güntert, 2004). Analysis of dihedral angles using the PROMOTIF program (Hutchinson and Thornton, 1996) indicates that in micelle solution composed by DPC/SDS at molar ratio 90:10 and 60:40 (Figure 2) the prevalent C8 conformation is a type I β-turn where the residue Val5 represents the center of this secondary structure element. The occurrence of this structure decreases at lower concentrations of the zwitterionic DPC phospholipid. Specifically, in micelle solutions composed by DPC/SDS 60:40, 45 out of 50 C8 conformers include type I β-turn on Trp4-Trp7; conversely, in DPC/SDS 40:60 molar ratio, type I β-turn is present in 23 out of 50 C8 conformers. In mixed 10:90 DPC/SDS micelles, C8 is in prevalent random coil conformations with a minor presence of γ-turn involving the Trp1-Asp3 residues (insets of Figures 2E,F). These results are consistent with those deriving from the previously published conformational study (Scrima et al., 2014). Analysis of side chains indicates that in micelle solutions characterized by a higher concentration of zwitterionic phospholipids, indolyl rings of Trp1 and Trp4 form an extended hydrophobic surface, likely favored by the type I β-turn structure assumed by C8.

### The C8-Membrane Surface Interaction: NMR and ESR Spectroscopy

Analysis of C8 chemical shifts in the different mixed micelle solutions indicates changes in NH signals, particularly those belonging to the N terminal residues (see Figure S3).

To identify the residues most involved in the interaction with lipid aggregates, we collected TOCSY spectra of C8 in DPC/SDS (90:10, 60:40, 40:60, and 10:90 molar ratio), in the presence and absence of 5-doxylstearic acid (5-DSA) and 16-doxylstearic acid (16-DSA) while keeping all other conditions constant (Solomon, 1955; Esposito et al., 2006).

5-DSA and 16-DSA are paramagnetic probes containing a doxyl head group with unpaired electrons that is bound to the aliphatic carbon chain at either position 5- or 16-. Paramagnetic probes are able to induce a broadening of the NMR signal and a decrease in the resonance intensities. Generally, the sites of the peptide that are closest to the NOE moiety are affected by the unpaired electrons, with an increase in nuclear relaxation rates and, thus, a decrease in proton signals. If the peptide is close to the surface, a decrease in intensity is observed in the 5-DSA spin-labeled micelles, but if the peptide penetrates the inner core of the micelle, a decrease in intensity is observed in the 16-DSA spin-labeled micelles (Mannhold et al., 2006).

Similarly to what we previously observed in pure DPC micelles (Scrima et al., 2014), 5-DSA in DPC/SDS 90:10 molar ratio (Figure S4) induces a dramatic effect on NH/CHα cross-peaks of Trp1, Trp4, and Gly6. In all the other conditions (60:40, 40:60, and 10:9 molar ratio) the change in signal intensity was in the range of the experimental error (Figure S4). Altogether these data indicate a prevalent exposition of the C8 N-terminal region to the micelle surface.

**Figure 3:**
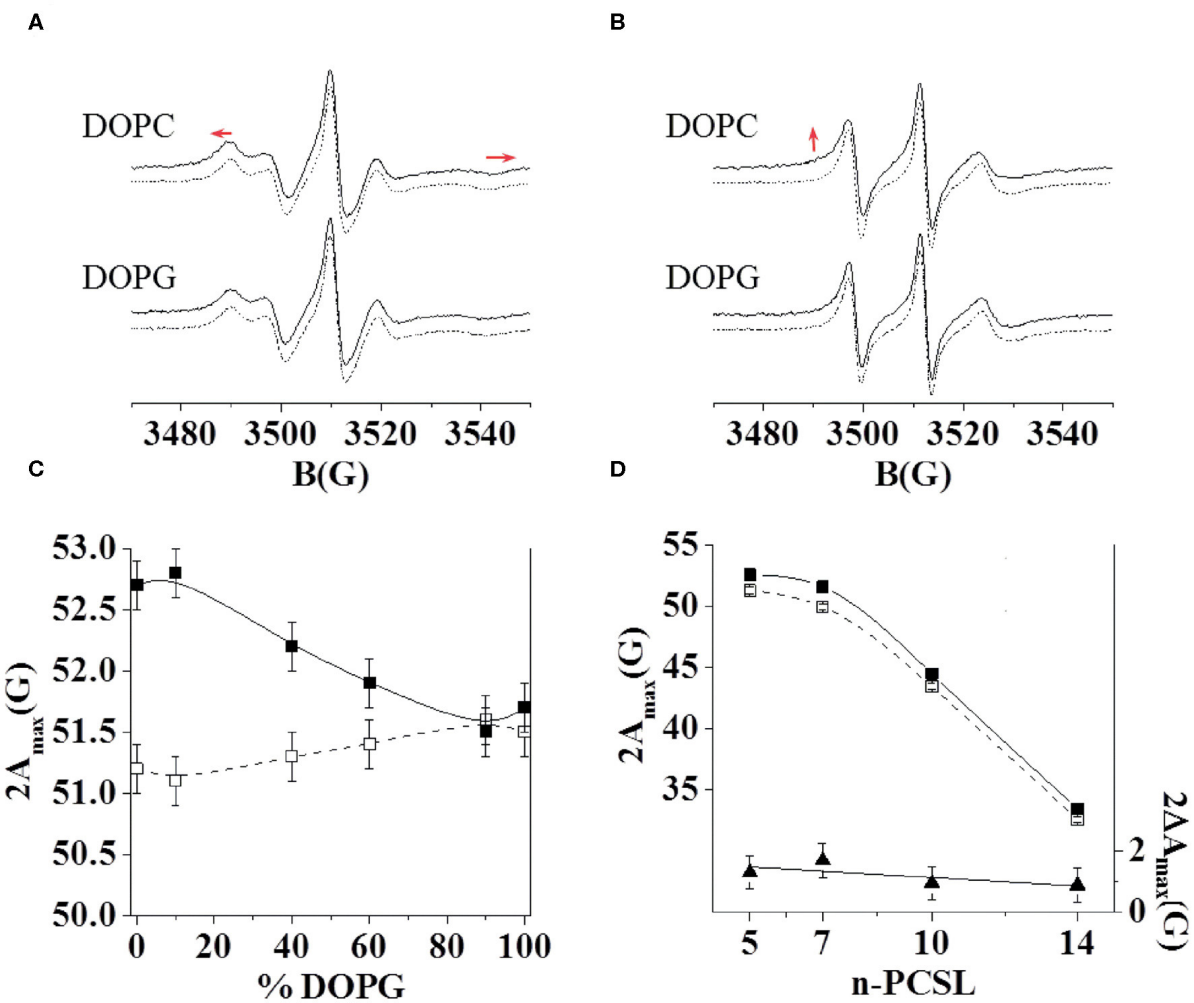
ESR spectra of 5-PCSL **(A)** and 14-PCSL **(B)** in lipid bilayers of DOPC and DOPG in the absence (dashed lines) and presence (continuous lines) of the C8 peptide. In **(A)** the red arrows highlight the shift of the low-field maximum and high-field minimum. In **(B)** the red arrow highlights the evidence of a second component in the spectrum registered in the presence of C8. 5-PCSL dependency on the DOPG percentage in the lipid bilayer composition of the outer hyperfine splitting, 2A_max_ **(C)**, in the absence (open squares, dashed line) and in the presence of the C8 peptide (solid squares, continuous line). n-PCSL dependency on spin-label position, n, of the outer hyperfine splitting, 2A_max_ (**D**, left-hand ordinate), in DOPC membranes in the absence (open squares, dashed line) and in the presence of the C8 peptide (solid squares, continuous line); to better highlight the 2A_max_ variation due to the presence of the peptide, the dependency on n of the difference between the 2A_max_ values in the presence of the C8 peptide and in its absence is also reported (**D**, right-hand ordinate, solid triangles).

Furthermore, we investigated the interaction of C8 with the zwitterionic DOPC, the negatively charged DOPG, and the mixed DOPC/DOPG bilayers through ESR experiments in order to reproduce experimental conditions similar to those employed for NMR analysis. In particular, we detected changes in the ESR spectra of spin-labeled lipids included in the membrane, precisely four phosphatidylcholines spin-labeled at different positions in the sn-2 chain, n-PCSL (*n* = 5, 7, 10, 14; Marsh and Horvath, 1998; Vitiello et al., 2010; Merlino et al., 2012; Bruno et al., 2015). The comparison between the results obtained with the different spin-labeled lipids allows a detailed description of the dynamics and the ordering of the lipids acyl chains in the whole bilayer profile. Firstly, we analyzed the effect of C8 on the 5-PCSL spectra in pure DOPC bilayers (Figure 3A). Both in the absence and presence of the peptide, the spectra show an anisotropic line shape, typical of labels inserted in layered supramolecular structures, detectable from the evident splitting of the low- and high-field signals. In the presence of the peptide, the anisotropic features become more evident, as highlighted by the evident shifts of the high-field maximum and low-field minimum. To quantitatively detect this effect, we measured the separation in gauss between these points, called 2A_max_, which is an index of restriction of the local mobility of the acyl chains. The 2A_max_ value of 5-PCSL in DOPC bilayers increases by 1.5 G in the presence of C8, revealing a stiffening of the outer region of the bilayer due to the peptide adsorption at the bilayer interface. Similar effects were observed for the same peptide interacting with bilayers formed by phosphocholine lipids with different acyl chains. In the case of dimyristoyl-phosphatidylcholine (DMPC), which bears two saturated acyl chains, a 2A_max_ increase of about 3.5 G was observed at temperatures at which the bilayer is in the liquid crystalline state, thus being fully comparable with the present results (D'Errico et al., 2009). For palmitoyl-oleoyl-phosphatidylcholine (POPC), presenting one saturated and one monounsaturated acyl chains, the 2A_max_ variation was about 2 G (Merlino et al., 2012). In the present work, we find a 1.5 G increase due to the C8 interaction with bilayers formed by DOPC, which bears two monounsaturated acyl chains. Thus, the perturbation due to the peptide decreases in more disordered and fluid membranes, remaining, however, clearly detectable.

Secondly, the effects of the peptide on the 14-PCSL spectrum in pure DOPC were considered (Figure 3B). In the absence of C8, an almost isotropic three-line shape is observed, due to the large motion freedom of the acyl chain termini in fluid lipid bilayers. In the peptide presence, the spectrum shows weak but significant changes: a shoulder can be observed at low field, while the high-field minimum is broadened, both pieces of evidence point to perturbation of the bilayer inner core due to the C8-bilayer interaction. We further investigated the C8 effects by also analyzing the spectra of 7-PCSL and 10-PCSL. The 2A_max_ variations along the entire lipid tail profile of DOPC are shown in Figure 3D. 2A_max_ increases by almost the same extent at all spin-label position, indicating that the entire membrane is significantly perturbed, in the direction of a lower bilayer fluidity and lipid dynamics.

Thirdly, we investigated, using the same approach, the C8 interaction with lipid bilayers formed by DOPG and DOPC/DOPG at molar ratio 90:10, 60:40, 40:60, and 10:90. In the case of the pure anionic bilayer, no effect of the peptide was observed, the spin-labels spectra remaining unperturbed (Figures 3A,B for the 5- and 14-PCSL spectra, respectively). This reflects in almost unchanged 2A_max_ values. Figure 3C shows the 2A_max_ value of 5-PCSL, in the absence and the presence of C8, as a function of the DOPG percentage in DOPC/DOPG mixed bilayers. This experimental evidence shows a gradual decrease in the observed lipid perturbation due to the interaction with C8 as the concentration of the anionic lipid increases. C8 can interact with bilayers containing a maximum of 60% DOPG, while in membrane with higher DOPG content, the interaction is lost.

### The C8 Binding Mechanism to Membrane: Funnel-Molecular Dynamics (FMD) Simulations

**Figure 4:**
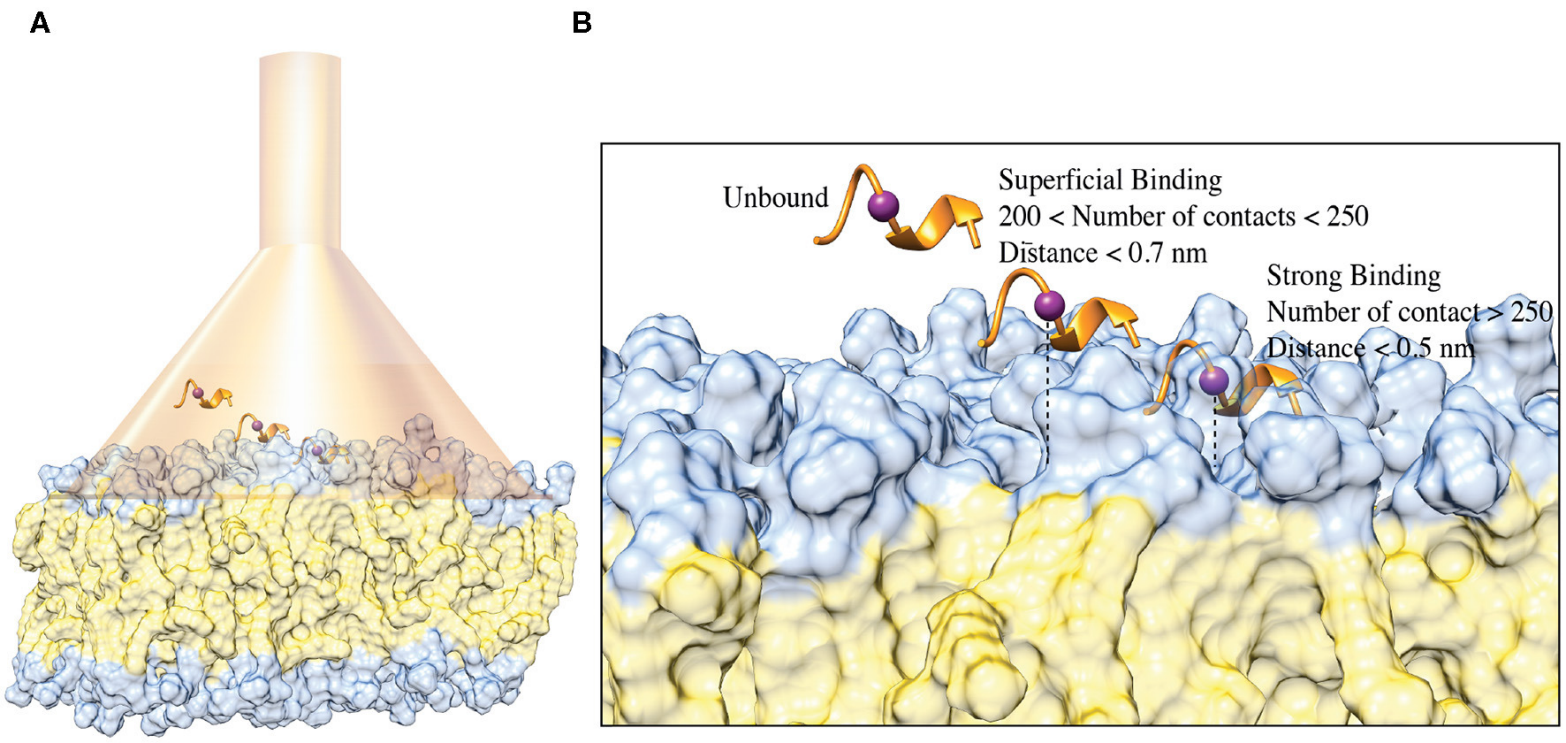
**(A)** Schematic representation of the FMD setting. C8 peptide is represented in the orange ribbon, while the purple spheres represent the Cα atom of Val5. The membrane is represented as a molecular surface where the polar heads and the aliphatic chains of the phospholipids are colored in light blue and in lithe yellow, respectively. **(B)** Schematic representation of the binding states of the C8 peptide with the membrane. The peptide and the membrane are represented as in **(A)**. The distance between Cα atom of the Val5, and the polar heads of the phospholipids is reported as a dashed line.

**Figure 5:**
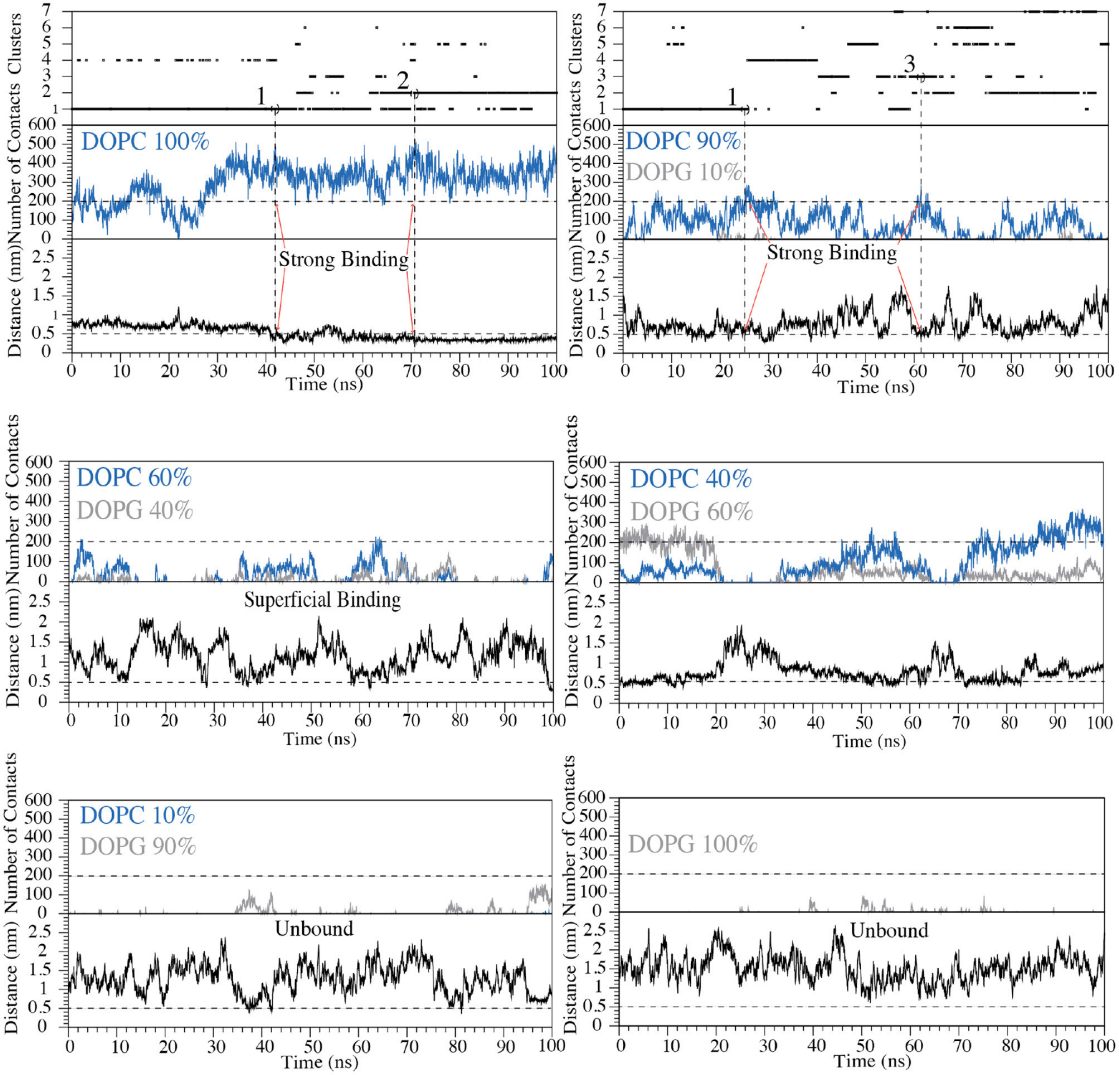
Cluster analysis, number of contacts and distance between Cα atom of Val5 and the polar heads of phospholipids are reported as a function of the simulation time for each of the six systems with the following zwitterionic/negatively charged phospholipids composition: pure DOPC, DOPC/DOPG 90:10, 60:40, 40:60, 10:90, and pure DOPG. The states representing “strong binding” modes in DOPC and DOPC/DOPG 90:10 are labeled by red arrows. The horizontal dashed line shows the cut-off value for the number of contacts between C8 and phospholipids used to distinguish the bound state from the unbound state of C8. The vertical dashed lines define the structures of the cluster families that were selected and animalized in comparison to the NMR structures.

Both the NMR and ESR data show a direct interaction of C8 with the membrane bilayers composed with a higher concentration of the zwitterionic DOPC phospholipids. In order to provide mechanistic insight into the peptide/membrane binding interaction, we performed atomistic molecular dynamics simulations, a well-established approach to study peptides functional and structural properties (D'Annessa et al., 2020), on C8 in six different membrane models composed by the same ratios of zwitterionic/negatively charged lipids used in the experiments. Specifically, they are (i) pure DOPC 100:0, (ii) DOPC/DOPG 90:10, (iii) 60:40, (iv) 40:60, (v) 10:90, and (vi) pure DOPG 0:100 (Figures 4, 5). One might note that the peptide/membrane binding process has typically long timescale, from μs to s, thus limiting the application of standard simulation techniques like classic MD. We overcame such limitation by performing FMD calculations in which a funnel-restrained potential is applied onto the system to considerably reduce the conformational space to explore in the unbound state (Figure 4A). As described by some of us in the original paper of the method (Limongelli et al., 2013), the funnel potential has a cone section that includes the binding region on the target molecule, here the membrane, while the cylinder section points toward the solvent. In such a way, during the simulation, the ligand, here C8, explores all the possible binding modes in the target structure, here the outer membrane leaflet, while the exploration of the unbound region is reduced to the cylinder section. This approach allows observing in a reasonable computational time a large number of binding and unbinding events between the ligand and the target molecule. It is also important to underline that when the ligand is inside the funnel (Figure 4A), no external potential is felt by the system, and the sampling proceeds as regular (no funnel) simulation. We have already successfully used FMD to study peptide/membrane binding interaction (Bruno et al., 2015).

In the present case, we performed FMD simulations to describe the binding mechanism of the peptide C8 to six membrane models, each of 49 nm^2^ composed by a different DOPC/DOPG ratio, as previously reported. The funnel potential is applied to the systems as shown in Figure 4A. We point out that the funnel-shape potential does not affect the peptide binding, provided that the funnel is wide enough to allow the peptide to explore a large surface of the membrane, as done in the present work. At the same time, the cone section favors the binding and unbinding in a reasonable computational time. The forth and back events between bound and unbound states are necessary to allow the peptide to explore alternative binding mechanisms and the membrane lipids rearranging when the peptide is not bound. The latter is particularly important in the case of non-homogenous membrane behavior as in the case of bilayers composed by different lipids (DOPC/DOPG 90:10, 60:40, 40:60, 10:90). Also, to avoid any effect in the selection of the peptide/membrane binding modes by the presence of the funnel potential, we filtered out all the bound states in which the system feels the funnel potential.

For each of the six systems we have performed 50 ns of preparatory classical MD simulation and then 100 ns of FMD using the type I β-turn structure of C8 resolved by NMR as a starting state (Figure 2). We decided to run preparatory simulations to ensure a proper equilibration of the lipid bilayer. Using FMD, the peptide (un)binds many times the membrane; therefore, the lipids could move and reorganize in the membrane plane in the presence and absence of C8 peptide.

We remark that the starting conformation of the peptide is not relevant in our calculations since C8 was fully flexible during the simulation and thus able to explore all the possible conformational states. At the end of the FMD calculations, we characterized the binding poses of C8 using the following two collective variables (CVs): (i) the number of contacts between the peptide and the polar heads of the phospholipids (CV1) and (ii) the minimum distance between the Cα atom of Val5 in C8 and the atoms of the polar heads of the phospholipids (CV2) (Figures 4B, 5). Using such CVs we could identify three different binding states of C8: (i) the unbound state (number of contacts <200 and distance >0.7 nm), (ii) a shallow binding state (200 < number of contacts <250 and distance <0.7 nm) and (iii) a deep binding state (number of contact >250 and distance <0.5 nm).

Our data show a stronger interaction of C8 with membranes composed by a higher concentration of DOPC rather than DOPG. In particular, in the pure DOPC system, C8 forms stable interactions, being CV1 significantly higher than 250 and CV2 lower than 0.5 nm for most of the simulation time (Figure 5). In the three systems with DOPC/DOPG 90:10, 60:40, and 40:60, C8 binds several times to the membrane (see Distance CV in Figure 5). However, the peptide/membrane number of contacts (CV1) is lower if compared with that calculated in pure DOPC. In all these cases, C8 mostly interacts with the zwitterionic lipid polar heads of the DOPC phospholipids rather than with the negatively charged heads of DOPG, as shown in Figure 5 (blue lines vs. gray lines, respectively). Finally, in systems with a higher concentration of DOPG (i.e., DOPC/DOPG 10:90 and pure DOPG), C8 remains in the unbound state for most of the simulation time, and no binding event occurs (Figure 5). This behavior is reflected by the low CV1 value in these systems that can be ascribed to the electrostatic repulsion effect between the negatively charged residues of C8 (Glu2 and Asp3) and the negatively charged polar heads of DOPG.

### Comparison of the *in*-*silico* and NMR C8 Structure

Our simulations are in agreement with the NMR and ESR data, further supporting the observation that C8 strongly interacts with membranes having a higher content of zwitterionic phospholipids (Figures 3, 5). We then characterized the structures of C8 in pure DOPC and DOPC/DOPG 90:10 membrane. For this purpose, the binding conformations assumed by the peptide during the FMD simulations in the two systems were clustered in families according to the root mean square displacement (RMSD) of the C8 backbone atoms (Figure 5). Specifically, conformations with RMSD values higher than 2 Å were assigned to different families. The representative structure of the most populated clusters was selected based on the highest and lowest value of CV1 and CV2, which are the maximum number of contacts and the minimum distance between C8 and the membrane, respectively (Figure 5).

In pure DOPC, two binding conformations of the peptide were identified from the most populated clusters, one representative of cluster 1 and the other of cluster 2 (Figure 5). We note that together clusters 1 and 2 represent ~85% of all the conformations assumed by the peptide in pure DOPC. Also, in DOPC/DOPG 90:10, two binding conformations were identified, one representative of cluster 1 and the other of cluster 3 (Figure 5). The second most populated family, cluster 2 in Figure 5 shows the peptide instead in a pre-binding state in which it establishes more superficial and weaker interactions with the membrane if compared with states in clusters 1 and 3.

**Figure 6:**
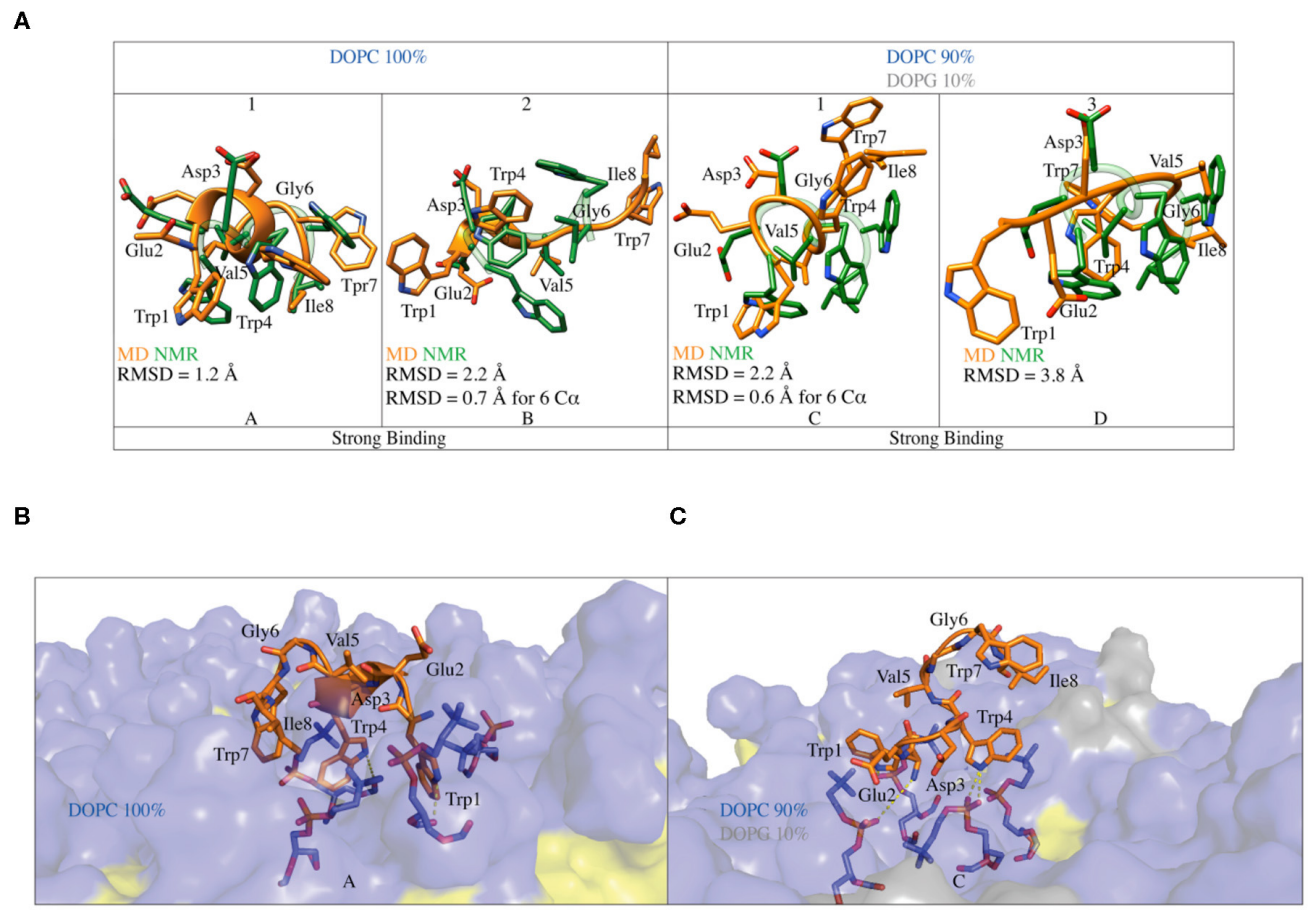
**(A)** Superimposition of the C8 binding conformation predicted by FMD (orange) with the NMR (green) structures obtained in pure DOPC and DOPC/DOPG 90:10. The peptide secondary structure is shown as a cartoon. The RMSD of the Cα atoms of each alignment is also reported. **(B)** Representation of the binding mode for the C8 peptide to pure DOPC membrane. Phospholipids are represented as transparent surface, aliphatic chains are colored in yellow, while polar heads are colored in light blue. The DOPC polar heads interacting with C8 are highlighted as sticks and the h-bonds as dashed lines. **(C)** Representation of the binding mode for the C8 peptide to DOPC/DOPG 90:10 membrane. The graphics is the same as in **(B)** with the addition that the polar heads of DOPG are colored in light gray.

For this reason, we do not further consider cluster 2 in our discussion. The peptide binding conformations representative of the most populated clusters in pure DOPC and DOPC/DOPG 90:10 were superimposed with the C8 NMR structures obtained in the equivalent phospholipid environment i.e., pure DOPC with pure DPC (Scrima et al., 2014); DOPC/DOPG 90:10 with DPC/SDS 90:10. Figure 6A shows that the peptide binding conformations obtained from the simulations in pure DOPC membrane are remarkably similar to the NMR structure obtained in pure DPC micelles. We assessed this similarity computing the RMSD of the Cα atoms in the binding conformation of cluster 1 and cluster 2 relative to the NMR structure. The value is 1.2 Å for the binding conformation A representative of cluster 1, while it is 2.2 Å for the binding conformation B representative of cluster 2 (Figure 6A). In the latter case, the RMSD becomes smaller (0.7 < Å) if considering the first 6 N-terminal residues, which are involved in the interaction with the membrane phospholipids according to our NMR and *in silico* data (see “The C8 structure: CD and NMR spectroscopy” paragraph). Furthermore, for these residues (the first 6 N-terminal residues) we computed a low RMSD value of 0.82 Å of the Cα atoms between the binding conformation A and B (see Figure S5), leading to the conclusion that one single binding mode of C8 can be considered in pure DOPC membrane. The higher population of cluster 1 prompted us to consider hereafter A as the C8 binding mode in pure DOPC.

A similar approach was used to compare the peptide binding conformations identified by FMD in DOPC/DOPG 90:10, with that found by NMR in the equivalent phospholipid environment. In this case, the RMSD calculated for the Cα atoms between the binding conformation C representative of cluster 1 and the NMR structure is 2.2 Å. Similarly to what found for cluster 2 in DOPC, this value becomes smaller (0.6 Å) when only the first 6 N-terminal residues of C8 are considered in the RMSD calculation (C in Figure 6A). At variance with pose C, the binding conformation D representative of cluster 3 is rather different from the NMR structure with an RMSD value of 3.8 Å (D in Figure 6A). The dissimilarity of this pose with the NMR structure, the lack of ordered structural motifs, together with the minor population of cluster 3, prompted us to discard this pose and hereafter consider C as the C8 binding mode in 90:10 DOPC/DOPG. A detailed structural description of the C8 binding mode in pure DOPC and 90:10 DOPC/DOPG (A and C in Figure 6A, respectively) is provided in the following paragraphs.

### The C8 Binding Mode

In the binding mode A in pure DOPC membrane, the C8 peptide interacts with the membrane surface through its tryptophan residues, which have their side chains oriented toward the phospholipids heads (Figure 6B). The three tryptophans residues are deepened in the membrane, establishing three cation-π interactions with the positively charged nitrogen atom of phosphatidylcholine groups (Figure 6B). The side chains of the tryptophan residues form several H-bonds with both carbonyl and phosphate oxygens of phosphatidylcholine groups, further stabilizing the C8/membrane interaction (Figures 5, 6B). The N-terminal region of the C8 peptide also contains two negatively charged amino acids, Glu2, and Asp3 that can form salt bridge interactions with the positively charged nitrogen atoms of the DOPC polar heads (see Figure S6A). All the described C8/membrane interactions are highly conserved among the states forming cluster 1 apart from those formed by Trp7 that assumes a more superficial position in the binding mode (see Figure S7A). Finally, it is worth noting that all the peptide conformations in cluster 1 and, consequently, the binding mode shown in Figure 6A, are characterized by a distance of <7 Å between the Cα atoms of residues i and i+3 stabilized by H-bonds such as Trp1-Trp4, Glu2-Val5, Val5- Ile8, and Trp4-Trp8, most of these described by CD and NMR as the hallmark of the type I β-turn structure.

Similarly to what found in pure DOPC, also in 90:10 DOPC/DOPG (i.e., binding mode C in Figure 6C), the interaction of the C8 peptide with the membrane is mediated by the tryptophan residues. In particular, Trp1 and Trp4 point to the membrane-forming three H-bonds and three cation-π interactions with the DOPC zwitterionic polar heads. At variance with the binding mode A, here, Trp7 is solvent-exposed and does not interact with the phospholipid atoms (Figure 6C). Comparably to what seen in pure DOPC, the negatively charged residues of C8, Glu2, and Asp3 can form electrostatic interactions with the phosphatidylcholine groups of DOPC (see Figure S6B). The binding mode C is mainly stabilized by the salt bridge and the cation-π interaction formed by Asp3 and Trp4 with the phospholipid polar heads, respectively. These are the most conserved peptide/membrane interactions among the states forming cluster 1 (see Figure S7B). Finally, the binding mode C has the Cα atoms of residues i and i+3 distant <7 Å and is stabilized by H-bonds such as Trp1-Trp4, Glu2-Val5, and Trp4-Ile8, characterizing a type I β-turn secondary structure. However, the absence of Trp4-Trp7 and Val5-Ile8 H-bond suggests higher flexibility of the C-terminal region of C8 (residues 6–8).

### The C8 Binding Free-Energy Profile

**Figure 7:**
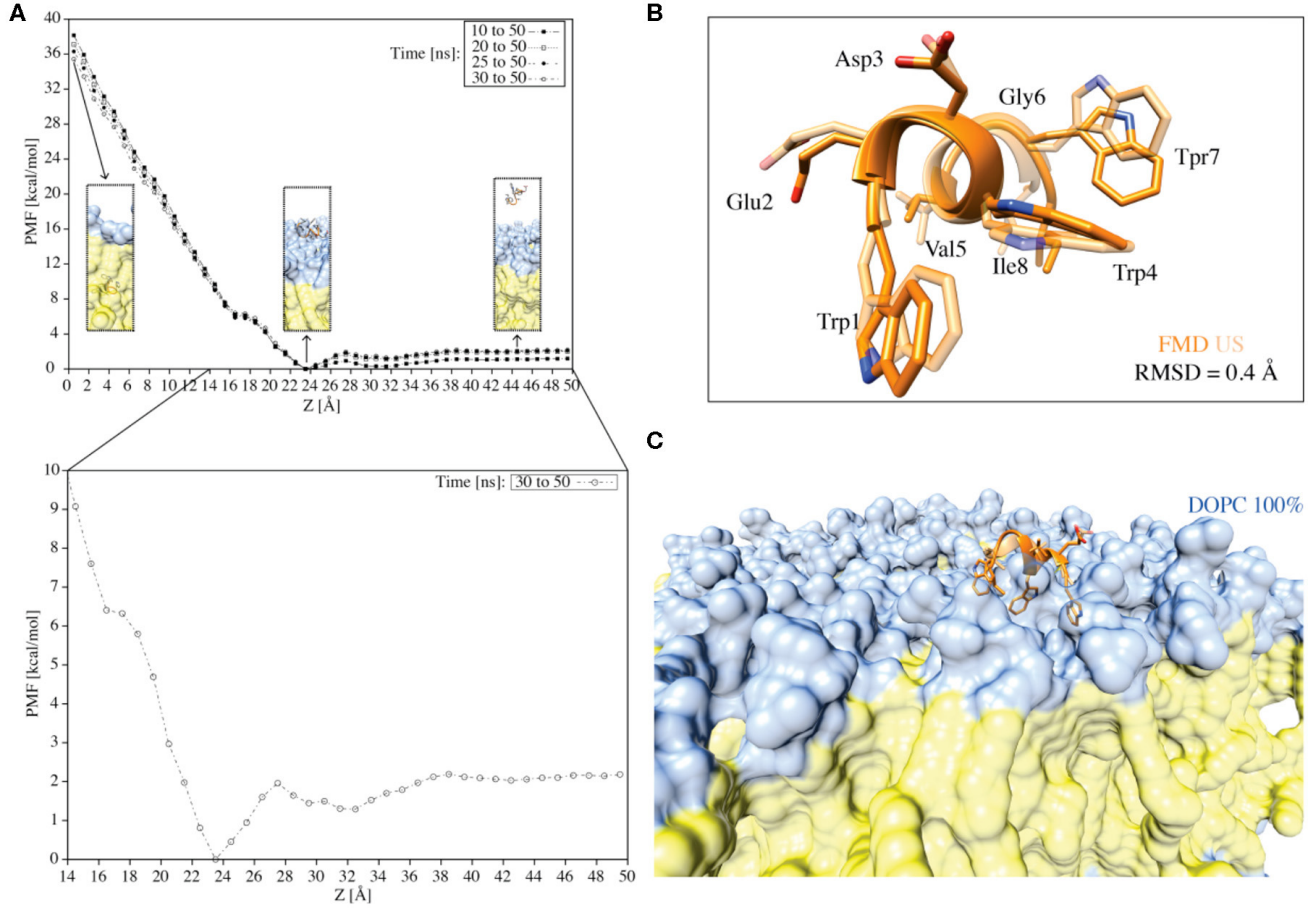
**(A)** The PMF for the translocation of the C8 peptide through a pure DOPC bilayer calculated using the umbrella integration technique. The different curves represent the PMFs computed over different simulation times. *Z* = 0.0 Å corresponds to the center of the bilayer. The inserts indicated by the arrows represent the peptide in the unbound state, the peptide in the membrane-bound state, and the peptide immersed into the membrane. The color code followed that reported in previous figures. The PMF for the translocation of the C8 peptide through a pure DOPC bilayer calculated in the last 20 ns of simulation (i.e., 30–50 ns) with Z values between 14 and 50 Å is also reported (i.e., the zoom insert). **(B)** Superimposition of the C8 peptide extracted at *Z* = 23 Å (i.e., the free-energy minimum) from the US (light orange) and the FMD (orange) simulations. **(C)** Visualization of the superposition depicted in B with the addition of the membrane.

The FMD simulations indicate that C8 binds the extracellular membrane leaflet without embedding in the bilayer. Other amphipathic peptides have shown a similar binding behavior with the membrane interface and then followed by the crossing of the membrane bilayer (Limongelli et al., 2013). One might note that FMD is a biased sampling method and the presence of the funnel potential might produce artifacts in the results. Therefore, it is recommended to validate the binding mode obtained from FMD by means of either unbiased MD (i.e., assessing the stability of the binding mode) or free-energy calculation methods (i.e., validating the binding mode as low energy bound state). Here, in order to provide a more in-depth structural and energetics insight into the peptide interaction with the phospholipid bilayer, we performed 2.5 μs of umbrella sampling (US) calculations on C8 in the pure DOPC membrane system (Figure 7A and Figure S8).

First, we generated 50 starting states (windows) in which the C8 COM is separated by 1 Å from one state to the other along the membrane axis Z. These states cover the whole binding process, from the fully solvated state (*Z* = 50.0 Å) to the peptide at the center of the membrane bilayer (*Z* = 0 Å). Then, we performed 50 ns of US calculations for each of the 50 windows, for a total of 2.5 μs (Figure 7A). The potential of mean force (PMF) was calculated using the Weighted Histogram Analysis Method (WHAM) (Kumar et al., 1992) and reported in Figure 7A. The calculation of the PMF as a function of different simulation time intervals shows the convergence of the calculation (Figure 7A). The maximum in the PMF corresponds to the state in which the C8 is located at the center of the membrane (i.e., the peptide is buried into the membrane) (*Z* = 0 Å), while the lowest energy minimum (*Z* = 23–25 Å) represents the state where C8 interacts with the extracellular phospholipids polar heads.

We further analyzed the PMF profile to elucidate in more detail the energetics of the C8/membrane interaction (Figure 7A inset). The lowest free-energy minimum corresponds to the state in which the peptide is at the bilayer-water interface, interacting preferentially with the polar heads of the DOPC phospholipids (Figure 7A inset). We note that there is a small energy barrier of ~2 kcal/mol between this energy minimum (i.e., the bound state) and the unbound state (Figure 7A inset). This energy barrier is likely due to the desolvation energy, which is the energy cost for the reorganization of the water molecules around the peptide and the phospholipid polar heads during the C8/membrane interaction. The small energy barrier that separates the unbound from the bound state explains the observation of diverse binding and unbinding events during the FMD simulations (Figure 5). Furthermore, there is a small kink in the PMF profile around 16 Å (Figure 7A inset) corresponding to the state in which the peptide interacts with the DOPC tails inducing a conformational rearrangement of the bilayer. On the other hand, the energy barrier for the complete penetration of the C8 into the membrane is ~36 kcal/mol suggesting that the peptide binds the membrane at the extracellular leaflet and its translocation across the membrane is energetically unfavorable.

Finally, we compared the lowest energy binding mode obtained from the US (*Z* = 23 Å in the PMF shown in Figure 7B) with that from the FMD (Figures 6A,B binding mode A). The two binding conformations are fully in agreement, as demonstrated by the very low RMSD value of 0.4 Å calculated on the C8 Cα atoms (Figure 7B). The superimposition of the two binding poses reported in Figure 7B also shows that the orientation of the residues side chains and the binding interaction with the phospholipids are very similar (Figures 7B,C). The remarkable agreement between the FMD and US results achieved at a sensitively different computational cost (150 ns and 2.5 μs, respectively), endorses FMD as a most valid technique to investigate the binding of molecules to the membrane bilayer in particular when the binding free energy surface does not present significant energy barriers as in the case of C8.

### Effect of C8 on the Phospholipid Bilayer

**Figure 8:**
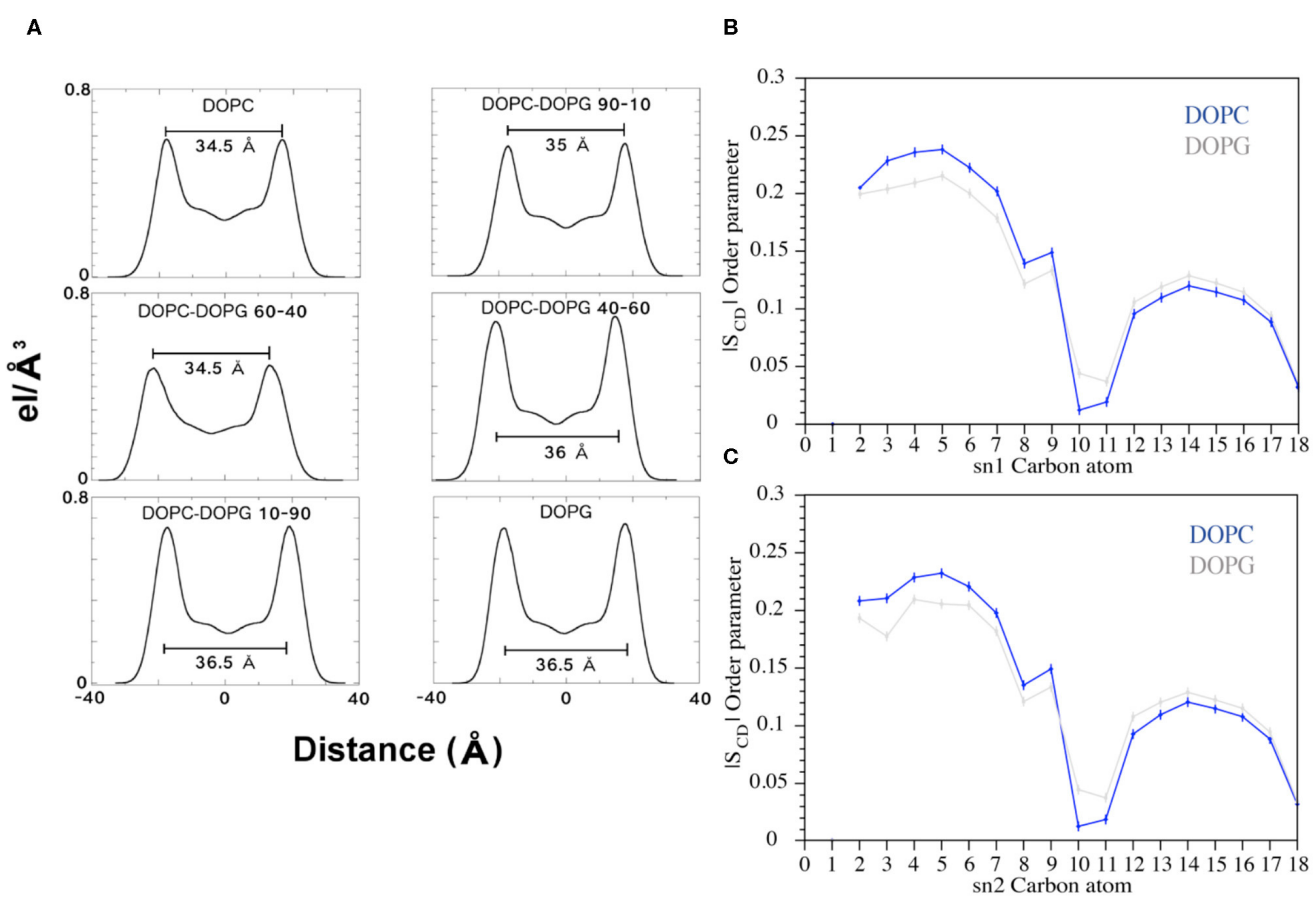
**(A)** Electron Density Profile (EDP) of the bilayers with different amounts of DOPC and DOPG phospholipids. For each system, the thickness was calculated (black bars) as the distance between the two peaks. **(B,C)** Plots of the SCD parameter computed for the sn1 and sn2 chains of the DOPC and DOPG phospholipids during the FMD simulations. The blue and gray lines represent DOPC and DOPG, respectively.

FMD and NMR data proved a C8-membrane binding mode mediated by exposure of the tryptophan residues to the apolar environment formed by the phospholipids. Furthermore, the ESR results showed a thinning of the membrane upon interaction with the peptide. Therefore, we decided to investigate the lipid perturbations upon the C8 by analyzing some physical properties of the membrane. We first looked at the thickness of the bilayer in all the six systems to evaluate its variation with growing percentages of the zwitterionic phospholipids (i.e., DOPC) in the membrane bilayer. The thickness of the membrane is computed as the average of the electron density along the bilayer computed over the total simulation time (see Experimental Section for details). Our results show that increased concentrations of the binding zwitterionic phospholipids (DOPC) are associated with the decreased thickness of the membrane, passing from 36.5 Å in pure DOPG to 34.5 Å in pure DOPC (Figure 8A). Also, computing the thickness of the DOPC bilayer in the US windows in which the peptide is in the unbound and bound state, we note that the bilayer becomes thinner upon the binding of the peptide passing from 36 to 34.5 Å. Based on these observations, we conclude that the higher the concentration of zwitterionic phospholipids, the stronger the C8/membrane interaction and the thinner the membrane is.

Furthermore, we analyzed the order parameter of the phospholipids, SCD, which estimates the organization of the lipids forming the membrane (Figures 8B,C). In particular, the higher the SCD value, the greater is the order degree of the lipids. In order to investigate the variation of the lipid organization related to the peptide binding, we compared the values of SCD computed in pure DOPC and pure DOPG. These indeed represent the two opposite cases, the former (DOPC) in which the peptide is bound to the membrane for most of the simulation time, and the latter (DOPG) in which no binding event is observed (Figure 5). The calculated SCD values agree with the experimental ones and with the computational data previously reported in the literature (Rosso and Gould, 2008; Dickson et al., 2014), and this result strongly supports that the C8 binding changes the organization of both the lipid carbon chains, sn1 and sn2, from the polar head to the fifteenth carbon atom (Figures 8B,C). Specifically, higher SCD values are observed in DOPC as compared to DOPG in the first fifth carbon atoms, while lower values are found after the tenth carbon. These data show that although the C8 binding occurs at the extracellular leaflet, it induces conformational rearrangements of the inner lipids that might lead to the remodeling of the bilayer. However, further investigations on larger systems employing longer timescale calculations are necessary to provide more insightful data in this sense.

### Change of the Membrane Morphology Upon C8 Binding: Confocal Microscopy Imaging

**Figure 9:**
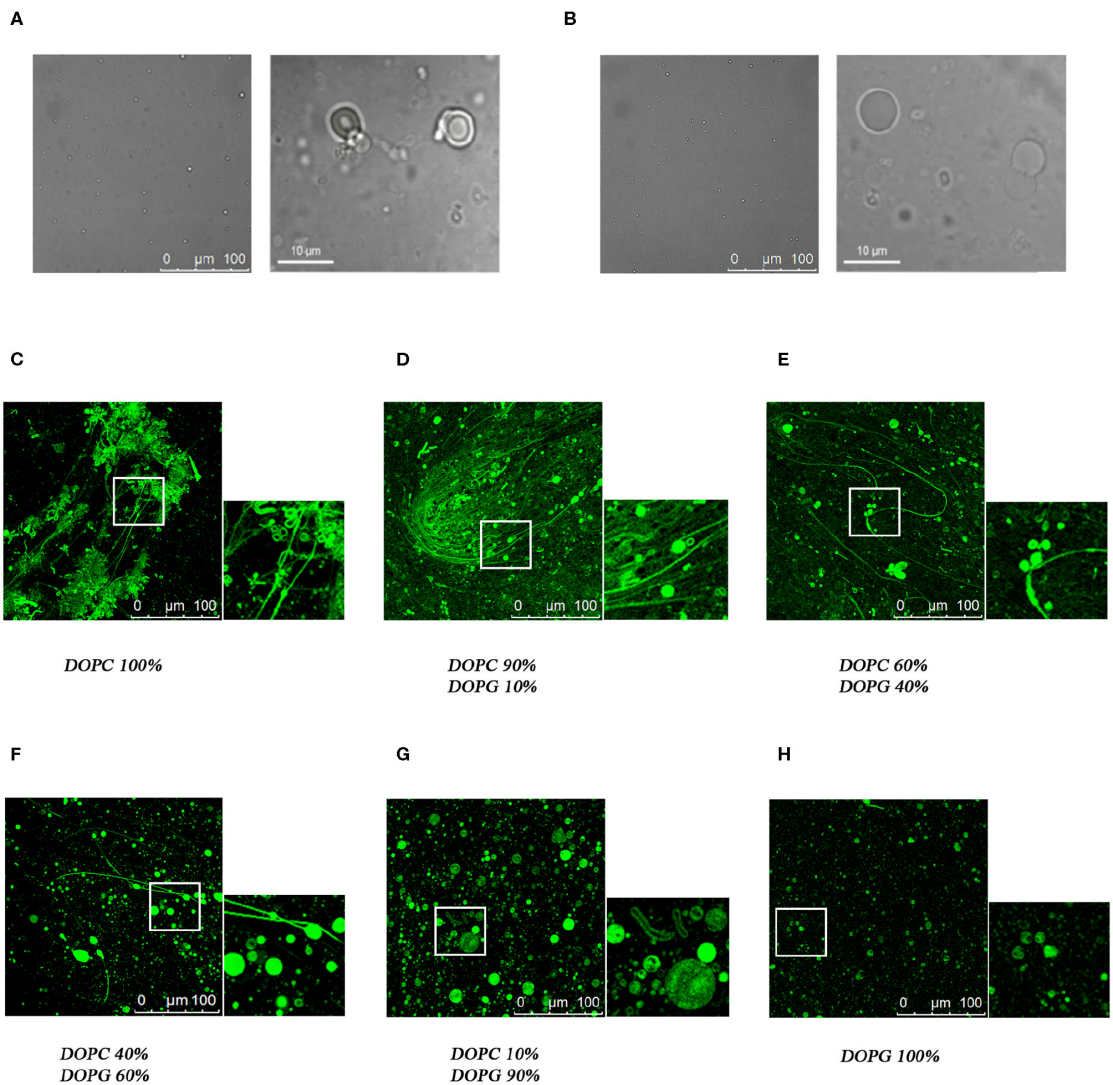
Confocal microscopy analysis of the effect of C8 peptide on MLVs membrane morphology. Representative images of MLVs in the absence **(A,B)** or in the presence **(C–H)** of 10% NBD labeled-C8 peptide. MLVs vary in DOPC/DOPG composition as reported in the labels.

Confocal microscopy has been extensively applied to investigate the membrane biophysical properties and the way these properties are modified by molecules with specific biological functions. We previously studied by confocal microscopy C8 and its shorter derivatives C6a and C6b using MLEVs of different DOPC/DOPG composition. These data showed that C8 and its derivative C6a is capable of modifying the curvature of MLVs by inducing membrane tubulation (Grimaldi et al., 2018). Figures 9A,B show that the MLVs assume a spherical architecture in the absence of peptide with a diameter ranging from 5 to 20 μm.

In the attempt to find a bridge between these observations and the atomistic scale description of the C8-lipid membrane interaction obtained in the present work from the NMR, ESR, and *in-silico* data, we repeated the confocal microscopy analysis, by including all the MLVs compositions consistent with the conditions employed in the present study. To this end, we collected confocal microscopy imaging using NBD labeled C8, named C8 (NBD1)W (Dufau and Mazarguil, 2000; Grimaldi et al., 2018), in MLVs composed of 40:60 and 60:40 molar ratios DOPC/DOPG respectively. These conditions are particularly significant, corresponding to a switch point from an excess of zwitterionic to an excess of negatively charged phospholipid. The confocal microscopy images shown in Figures 9E,F confirms that the peptide localizes on the membrane surface. In particular, MLVs composed of DOPC/DOPG 40:60 show a diameter ranging from 20 to 40 μM (Figure 9F). By comparing these dimensions to those reported for C8 in pure DOPG MLVs (Figure 9C; Grimaldi et al., 2018), it is evident the ability of C8 to induce membrane fusion. Figure 9E, displaying the effect of C8 on MLVs composed of DOPC/DOPG 60/40 molar ratio, indicates that in these conditions in addition to the vesicle fusion, C8 induces modification of the membrane shape with the formation of membrane “nipples” (Roux et al., 2002).

## Discussion

Increasing evidence shows that during the fusion of the virus with the host cell membrane, translational diffusion of the lipids induces a change in the bilayer composition (Chernomordik and Kozlov, 2008). In particular, a slight change in membrane charge affects the structure and function of those regions of the viral envelope glycoprotein, responsible for interaction with lipid membranes. Gp36 is the FIV envelope glycoproteins catalyzing the fusion of the virus with the host cells. We previously studied C8, the fragment corresponding to ^770^W-I^777^ of gp36 (Figure 1A; Lombardi et al., 1996; Giannecchini et al., 2003), in lipid vesicles of different compositions, and in micelle solution composed of negatively charged SDS, or zwitterionic DPC detergents (Scrima et al., 2014). To extend this characterization, in the present work we have investigated the effect induced by systematic variation of phospholipid charge on (i) the aptitude of C8 to interact with lipid membranes, (ii) the tendency of C8 to assume specific conformational states and (iii) the re-organization of the lipid bilayer upon the interaction with C8.

CD and NMR experiments show that in zwitterionic micelle solution C8 is present in regular β-turn structures that are progressively reduced as the concentration of negatively charged SDS (Figure 1B) or DOPG (Figure 1C) increases. In particular, in DPC/SDS 90:10, whose surface charge is considered representative of a biological membrane surface (Yue et al., 2014; Moltedo et al., 2019), C8 conformations are similar to those found in pure DPC micelles (Figure 1B; Scrima et al., 2014), suggesting that the presence of small quantities of negatively charged phospholipids slightly affects the structural stability of the C8 peptide. However, a clear reduction in the content of β-turn is observable when moving from DPC/SDS 60:40 to DPC/SDS 40:60, indicating that a slight excess of SDS detergent affects the tendency of C8 to assume regular secondary structures (Figure 1B).

Extensive theoretical calculations supported by ESR spectroscopy experiments show that the prevalence of ordered β-turn conformations in systems characterized by the prevalence of zwitterionic phospholipids is associated with a strong affinity of C8 for the lipid membrane. MD simulations carried out on six different DOPC membranes, each including increasing content of DOPG phospholipid, put in evidence the higher binding affinity of C8 for lipid bilayers enriched with zwitterionic phospholipids (Figure 5).

As all the experimental data converged on the aptitude of C8 to bind the zwitterionic lipid membrane, we turned to collect NMR spin aided experiments to predict the topology of C8 at the membrane interface. NMR spectra acquired in the presence of 5-doxyl stearic acid, supported by classical MD simulations and US calculations, show that C8 interacts with the outer membrane leaflet through Trp1 and Trp4 that establish cation-π and H-bond interactions with the DOPC zwitterionic polar head. Also, salt bridge interactions established between the negatively charged amino acids of C8 (i.e., Glu2 and Asp3) and the phospholipid zwitterionic polar heads contribute to stabilizing the interaction between the membrane and the peptide.

Peptide/protein-membrane interaction has been extensively studied, indicating that the binding of a peptide sequence with the lipid membrane occurs through different types of chemical interaction: cation-π, H-bond, hydrophobic interactions (Muller et al., 2019). Our data prove that in Trp rich peptide, like C8, Trp residues have the unique, versatile molecular properties to engage all these interactions simultaneously. Indeed Trp is the most polarizable residue that exhibits the largest non-polar surface area; it possesses an indole N-H moiety capable of hydrogen bond donation and displays the greatest electrostatic potential for cation-π interactions (Gallivan and Dougherty, 1999; Babakhani et al., 2007; Sanchez et al., 2011). In other cases, the complex role exerted by Trp may be played by a peptide sequence carrying a lipid-like anchor, as is the case of Ras GTPases and Rab5 GTPase (Muller et al., 2019). Computational studies have shown that interaction with phospholipid bilayer, for Ras and Rab5 GTPase is mediated by the chemical moieties of the amino acids. These are responsible for hydrogen bonding and electrostatic interactions; on the other hand, lipid side chains engage hydrophobic, van der Waals contacts, which stabilize protein-membrane binding (Gorfe et al., 2004; Edler et al., 2017).

Once we established (i) the aptitude of C8 to bind lipid membranes according to their charge content, once we clarified the effects exerted from this binding on C8 conformation and the molecular mechanism subtending the C8-membrane interaction, we decided to investigate the effect exerted by the peptide on the bilayer organization. ESR and computational data show that although adsorption of C8 onto the bilayer surface induces stiffening of the outer region of the lipid membrane, the perturbation in the bilayer organization is sensed on the entire membrane: (i) ESR experiments using four phosphatidylcholines spin-labeled at different positions in the sn-2 chain, n-PCSL (*n* = 5, 7, 10, 14; Figure 3), reveal 2A_max_ variations along the entire lipid tail profile of DOPC; (ii) *in silico* data show that the phospholipids order parameter, —that estimates the organization of the lipids forming the membrane (Figure 8B), decreases from the polar head until the fifteenth carbon atom (Figure 8B). (iii) US calculations show a kink in the potential of mean force profile at 16 Å, typical of a conformational rearrangement of the bilayer. ESR measurements and FMD data show that this bilayer rearrangement results in a remodeling of the bilayer as evident from the change in the bilayer thickness that decreases from 36.5 to 34.5 Å in pure DOPC (Figure 8A).

Using confocal microscopy imaging, we previously studied C8 in comparison with its shorter fragments C6a and C6b, on MLEVs of different DOPC/DOPG compositions (Grimaldi et al., 2018). These data showed that MLVs composed of pure DOPC or DOPC/DOPG 90:10 undergo a surface curvature modification upon the interaction of C8, resulting in a dense network of membrane tubes. This effect was not observed in MLVs composed of pure DOPC or DOPC/DOPG 10:90 in which the circular shape of the vesicles remains unmodified (Figure 9; Grimaldi et al., 2018). The data presently reported, suggest an interpretation at the atomistic level of what we observed at micron-scale in the mentioned microscopy investigation. Moreover, in the present work, we have studied C8 with confocal microscopy on MLVs composed of DOPC/DOPG 60:40 and 40:60, respectively. All the analytical and computational methods employed by us in the present study point to these conditions as critical to detect the change induced by different charge content on the conformational stability of C8 and on its affinity for the membrane surface. Confocal microscopy images shown in Figure 9 confirm that with the first excess of zwitterionic DOPC phospholipid, (i.e., 60:40 molar ratio), C8 binds and perturbs phospholipid bilayer to induce modification of the membrane shape with the formation of membrane “nipples.”

The discovery of the formation of membrane tubules induced by an antiviral membrane-binding peptide raises important questions such as (i) is the C8-induced membrane tubulation related to its antiviral activity? (ii) is the C8 able to play a destabilizing effect also on the virus envelope architecture affecting its virulence? (iii) are peptides endowed with anti-HIV (the human variant of the virus) activity also able to induce membrane tubulation? (iv) how could we control and exploit the peptide's ability to induce membrane tubulation in the drug design of new antiviral agents? The answers to these questions very likely pave the way to novel research scenarios unexplored so far.

Taking together our findings, it is tempting suggesting the C8 binding mode identified by the spectroscopic experiments and the atomistic simulations as the onset mechanism of the membrane remodeling that finally leads to membrane curvature, fusion, and tubulation as observed at confocal microscopy. This suggestive hypothesis requires, however, further investigations to achieve more insightful data able to investigate collateral factors that might play a role in the formation of membrane tubules. For instance, peptide concentration and consequently, peptide aggregation might influence the membrane curvature and then its tubulation. This kind of investigation is out of the scope of the present work and would require the employment of techniques different from atomistic simulations. In fact, the increased complexity of larger membrane systems and the slowly evolving dynamics of peptides in membrane require the employment of multiscale simulation protocols like coarse-grained MD, which has been successfully used to study large scale motion of peptides and proteins in the membrane (Lelimousin et al., 2016; Huber et al., 2019; Pannuzzo et al., 2019).

In conclusion, the present study is a successful example of a bottom-up multiscale approach capable of elucidating the structural and chemical properties governing the binding of a peptide to the lipid membrane. This information is necessary if one aims at controlling the lipid architecture rationally in complex processes like membrane fusion and tubule formation, which remain rather unexplored events despite their relevance in cell trafficking and cell communication processes (Yue et al., 2014; Moltedo et al., 2019). Our study has potential implications in the rational design of novel peptides with a controlled affinity toward the cell membrane. Having such control in peptide design can be strategically relevant to develop successful antiviral peptides, as well as in pharmaceutical technology to design “smart” peptides able to form engineered lipid architectures (e.g., vesicular structures and lipid-based bio-nanotubes) useful for molecular storage and dispenser of chemicals and drugs into living cells (Ariga et al., 2016).

## Data Availability Statement

All datasets generated for this study are included in the article/Supplementary Material.

## Author Contributions

DD, AB, AR, GB, and VL carried out the Funnel Molecular Dynamics (FMD) and the Umbrella Sampling simulations also performing all the analyses of the trajectories. MG and IS performed peptide SPPS and CD experiments. MG, MS, and AD'U performed NMR experiments and peptide structure calculation. IS, GA, GD, OM, and PR performed microscopy imaging experiments. GD'E performed ESR experiments. VL and AD'U designed the research and analyzed the data. All authors wrote and reviewed the manuscript.

## Conflict of Interest

The authors declare that the research was conducted in the absence of any commercial or financial relationships that could be construed as a potential conflict of interest.
