# Supplementary material for: Binding of the Anti-FIV Peptide C8 to Differently Charged Membrane Models: From First Docking to Membrane Tubulation
Source: Front Chem. 2020 Jun 26;8:493. doi: 10.3389/fchem.2020.00493 (PMC7333769; doi:10.3389/fchem.2020.00493)
Supplement: Supplementary file 1 [file Table_1.docx]

Supplementary Material

Binding of the anti-FIV peptide C8 to differently charged membrane models: from first docking to membrane tubulation

Daniele Di Marino,^‡1^ Agostino Bruno,^‡2^ Manuela Grimaldi,^‡3^ Mario Scrima,^3^ Ilaria Stillitano,^3^ Giuseppina Amodio,^4^ Grazia Della Sala,^5^ Alice Romagnoli,^1^ Augusta De Santis,^6^ Ornella Moltedo,^3^ Paolo Remondelli,^4^ Giovanni Boccia,^4^ Gerardino D’Errico,^6^ Anna Maria D’Ursi,*^3^ and Vittorio Limongelli,*^2,7^

^1^Department of Life and Environmental Sciences, New York-Marche Structural Biology Center (NY-MaSBiC), Polytechnic University of Marche, Ancona, Italy.

^2^Department of Pharmacy, University of Naples “Federico II,” via D. Montesano, 49, I-80131 Naples (Italy).

^3^Department of Pharmacy, University of Salerno, Via Giovanni Paolo II, 132, I-84084 Fisciano (Italy).

^4^Department of Medicine, Surgery and Dentistry "Scuola Medica Salernitana", University of Salerno, Via S. Allende, I-84081 Baronissi (Italy).

^5^Department of Neuroscience, Psychology, Drug Research and Child Health, University of Florence, 50139 Florence (Italy).

^6^Department of Chemical Science, University of Naples “Federico II,” via Cinthia, 80126 Naples (Italy).

^7^Faculty of Biomedical Sciences, Institute of Computational Science, Università della Svizzera italiana (USI), Via G. Buffi 13, CH-6900 Lugano (Switzerland).

To whom Correspondence should be addressed.

Prof. Anna Maria D’Ursi: dursi@unisa.it

Prof. Dr. Vittorio Limongelli: [vittoriolimongelli@gmail.com](mailto:vittoriolimongelli@gmail.com),

‡ equal contribution

**Figure S1.** Chemical structure of surfactants and phospholipids: a) dodecylphosphocoline (DPC), b) sodium dodecyl sulphate (SDS), c) 1,2-dioleoyl-sn-glycero-3-phosphocholine (DOPC) and d) 1,2-dioleoyl-sn-glycero-3-phospho-(1'-rac-glycerol) (DOPG).

**
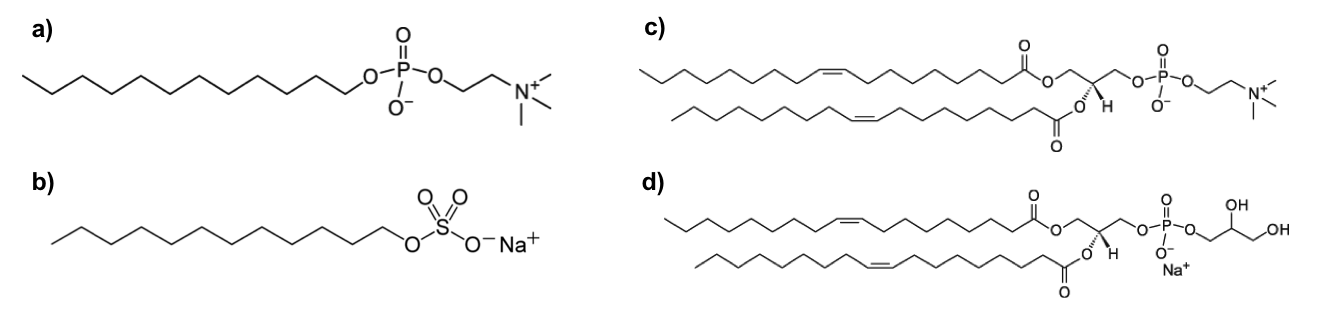
**

**Table S1.** Number of lipid molecules in each FMD system

| SYSTEM | Number of DOPC molecules | Number of DOPG molecules |
| --- | --- | --- |
| Pure DOPC | 254 | 0 |
| DOPC90%-DOPC10% | 229 | 25 |
| DOPC60%-DOPG40% | 152 | 102 |
| DOPC40%-DOPG60% | 102 | 152 |
| DOPC10%-DOPG90% | 25 | 229 |
| Pure DOPG | 0 | 254 |

**Table S2.** Proton chemical shift of C8 in DPC 100:0 M:M micelle solution H_2_O/D_2_O at 300 K

| **Residue** | **HN** | **CαH** | **CβH** | **C****γH** | **C****δH** | **C****εH** | **Others** |
| --- | --- | --- | --- | --- | --- | --- | --- |
| **Trp1** | 8.611 | 4.435 | Qβ 3.308 |  | Hδ1 7.416 | Hε1 10.667  Hε3 7.637 | Hη2 7.073  Hζ2 7.553  Hζ3 6.905 |
| **Glu2** | 9.176 | 3.886 | Hβ2 1.999  Hβ3 1.941 | Qγ 2.175 |  |  |  |
| **Asp3** | 8.046 | 4.505 | Hβ2 2.621  Hβ3 2.487 |  |  |  |  |
| **Trp4** | 8.040 | 4.373 | Qβ 3.415 |  | Hδ1 7.393 | Hε1 10.641  Hε3 7.548 | Hη2 7.027  Hζ2 7.480  Hζ3 6.788 |
| **Val5** | 8.093 | 3.914 | 2.155 | QQγ 0.989 |  |  |  |
| **Gly6** | 8.176 | Hα2 3.919  Hα3 3.955 |  |  |  |  |  |
| **Trp7** | 7.507 | 4.449 | Hβ2 3.435  Hβ3 3.387 |  | Hδ1 7.362 | Hε1 10.480  Hε3 7.559 | Hη2 7.120  Hζ2 7.506  Hζ3 7.035 |
| **Ile8** | 7.635 | 3.605 | 1.875 | Qγ12 1.544  Qγ13 1.104  Qγ2 0.957 |  |  |  |

**Table S3.** Proton chemical shift of C8 in DPC/SDS 90:10 M:M micelle solution H_2_O/D_2_O at 300 K.

| **Residue** | **HN** | **CαH** | **CβH** | **CγH** | **CδH** | **CεH** | **Others** |
| --- | --- | --- | --- | --- | --- | --- | --- |
| **Trp1** | 8.513 | 4.388 | Qβ 3.283 |  | Hδ1 7.432 | Hε1 10.522 | Hη2 7.094  Hζ2 7.549  Hζ3 6.918 |
| **Glu2** | 8.708 | 3.874 | Hβ2 1.911  Hβ3 1.833 | Hγ2 2.148  Hγ3 2.003 |  |  |  |
| **Asp3** | 7.992 | 4.487 | Hβ2 2.673  Hβ3 2.555 |  |  |  |  |
| **Trp4** | 7.970 | 4.374 | Qβ 3.407 |  | Hδ1 7.361 | Hε1 10.512  Hε3 7.530 | Hη2 7.030  Hζ2 7.419  Hζ3 6.805 |
| **Val5** | 7.952 | 3.894 | 2.152 | QQγ 0.980 |  |  |  |
| **Gly6** | 8.100 | Qα 3.913 |  |  |  |  |  |
| **Trp7** | 7.651 | 4.481 | Qβ 3.393 |  | Hδ1 7.340 | Hε1 10.386  Hε3 7.569 | Hη2 7.117  Hζ2 7.493  Hζ3 7.026 |
| **Ile8** | 7.556 | 3.644 | 1.834 | Qγ1 1.258  Qγ2 0.907 | Qδ1 0.871 |  |  |

**Table S4.** Proton chemical shift of C8 in DPC/SDS 60:40 M:M micelle solution H_2_O/D_2_O at 300 K.

| **Residue** | **HN** | **CαH** | **CβH** | **CγH** | **CδH** | **CεH** | **Others** |
| --- | --- | --- | --- | --- | --- | --- | --- |
| **Trp1** | 8.240 | 4.273 | Hβ2 3.219  Hβ3 3.187 |  |  | Hε1 10.322 | Hζ2 7.362  Hζ3 7.233 |
| **Glu2** | 8.209 | 3.773 | Hβ2 1.799  Hβ3 1.682 | Hγ2 2.049  Hγ3 1.885 |  |  |  |
| **Asp3** | 7.944 | 4.45 | Hβ2 2.722  Hβ3 2.654 |  |  |  |  |
| **Trp4** | 7.840 | 4.346 | Qβ 3.329 |  |  | Hε1 10.259 | Hζ2 7.449  Hζ3 7.233 |
| **Val5** | 7.680 | 3.845 | 2.061 | Qγ1 0.906  Qγ2 0.883 |  |  |  |
| **Gly6** | 7.974 | Qα 3.839 |  |  |  |  |  |
| **Trp7** | 7.622 | 4.459 | Qβ 3.323 |  |  | Hε1 10.222  Hε3 7.490 | Hζ2 7.441  Hζ3 7.256 |
| **Ile8** | 7.446 | 3.694 | 1.747 | Qγ1 1.334  Qγ2 0.966 | Hδ11 1 0.825  Hδ12 2 0.793 |  |  |

**Table S5.** Proton chemical shift of C8 in DPC/SDS 40:60 M:M micelle solution H_2_O/D_2_O at 300 K.

| **Residue** | **HN** | **CαH** | **CβH** | **CγH** | **CδH** | **CεH** | **Others** |
| --- | --- | --- | --- | --- | --- | --- | --- |
| **Trp1** | 8.027 | 4.231 | Hβ2 3.225  Hβ3 3.146 |  | Hδ1 7.366 | Hε1 10.155 | Hη2 7.344  Hζ2 7.424  Hζ3 7.200 |
| **Glu2** | 7.990 | 3.716 | Hβ2 1.756  Hβ3 1.604 | Qγ 2.003 |  |  |  |
| **Asp3** | 7.897 | 4.450 | Qβ 2.707 |  |  |  |  |
| **Trp4** | 7.793 | 4.385 | Qβ 3.325 |  | Hδ1 7.441 | Hε1 10.083 | Hη2 7.425  Hζ3 7.200 |
| **Val5** | 7.590 | 3.857 | 2.047 | Qγ1 0.899  Qγ2 0.870 |  |  |  |
| **Gly6** | 7.937 | Qα 3.844 |  |  |  |  |  |
| **Trp7** | 7.632 | 4.478 | Hβ2 3.341  Hβ3 3.320 |  | Hδ1 7.492 | Hε1 10.083 | Hη2 7.479  Hζ3 7.248 |
| **Ile8** | 7.425 | 3.751 | 1.739 | Qγ1 1.292  Qγ2 0.952 | Hδ11 0.812  Hδ12 0.783 |  |  |

**Table S6.** Proton chemical shift of C8 in DPC/SDS 10:90 M:M micelle solution H_2_O/D_2_O at 300 K.

| **Residue** | **HN** | **CαH** | **CβH** | **CγH** | **CδH** | **CεH** | **Others** |
| --- | --- | --- | --- | --- | --- | --- | --- |
| **Trp1** | 7.663 | 4.022 | Hβ2 3.075  Hβ3 2.942 |  | Hδ1 7.143 | Hε1 9.795 | Hζ2 7.245  Hζ3 7.023 |
| **Glu2** | 8.191 | 3.464 | Hβ2 1.376  Hβ3 1.314 | Hγ2 1.628  Hγ3 1.494 |  |  |  |
| **Asp3** | 7.601 | 4.254 | Qβ 2.305 |  |  |  |  |
| **Trp4** | 7.689 | 4.281 | Hβ2 3.189  Hβ3 3.155 |  | Hδ1 7.090 | Hε1 9.826 | Hζ2 7.245  Hζ3 7.022 |
| **Val5** | 7.600 | 3.734 | 1.908 | Qγ1 0.796  Qγ2 0.745 |  |  |  |
| **Gly6** | 7.786 | Qα 3.659 |  |  |  |  |  |
| **Trp7** | 7.507 | 4.246 | Hβ2 3.204  Hβ3 3.165 |  | Hδ1 7.235 | Hε1 9.765  Hε3 7.323 | Hζ2 7.294  Hζ3 7.090 |
| **Ile8** | 7.297 | 3.538 | 1.573 | Qγ1 1.120  Qγ2 0.745 | Hδ11 0.659  Hδ12 0.622 |  |  |

**Table S7.** Proton chemical shift of C8 in DPC/SDS 90:10 M:M micelle solution H_2_O/D_2_O at 300 K

| **Residue** | **HN** | **CαH** | **CβH** | **CγH** | **CδH** | **CεH** | **Others** |
| --- | --- | --- | --- | --- | --- | --- | --- |
| **Trp1** | 7.713 | 4.252 | Hβ2 3.303  Hβ3 3.142 |  | Hδ1 7.352 | Hε1 9.917  Hε3 7.451 | Hη2 7.124  Hζ2 7.407  Hζ3 6.945 |
| **Glu2** | 8.379 | 3.707 | Hβ2 1.616  Hβ3 1.549 | Hγ2 1.873  Hγ3 1.718 |  |  |  |
| **Asp3** | 7.770 | 4.476 | Qβ 2.520 |  |  |  |  |
| **Trp4** | 7.884 | 4.581 | Hβ2 3.424  Hβ3 3.367 |  | Hδ1 7.241 | Hε1 9.947  Hε3 7.576 | Hη2 7.170  Hζ2 7.473  Hζ3 7.051 |
| **Val5** | 7.752 | 3.992 | 2.142 | Qγ1 1.038  Qγ2 0.995 |  |  |  |
| **Gly6** | 7.966 | Qα 3.900 |  |  |  |  |  |
| **Trp7** | 7.731 | 4.483 | Hβ2 3.476  Hβ3 3.399 |  | Hδ1 7.340 | Hε1 9.887  Hε3 7.574 | Hη2 7.141  Hζ2 7.492  Hζ3 6.873 |
| **Ile8** | 7.454 | 3.826 | 1.813 | Qγ1 1.343  Qγ2 0.995 | Qδ1 0.895 |  |  |

**Figure S2.** Sequential and medium-range NOEs for C8 in DPC/SDS (H_2_O/D_2_O buffer) 100:0 (A), 90:10 (B), 60:40 (C), 40:60 (D), 10:90 (E) and 0:100 (F) molar ratios. Data were obtained from a 600 MHz NOESY experiments with a mixing time of 250 ms and collected at 300 K. (Scrima et al., 2014)


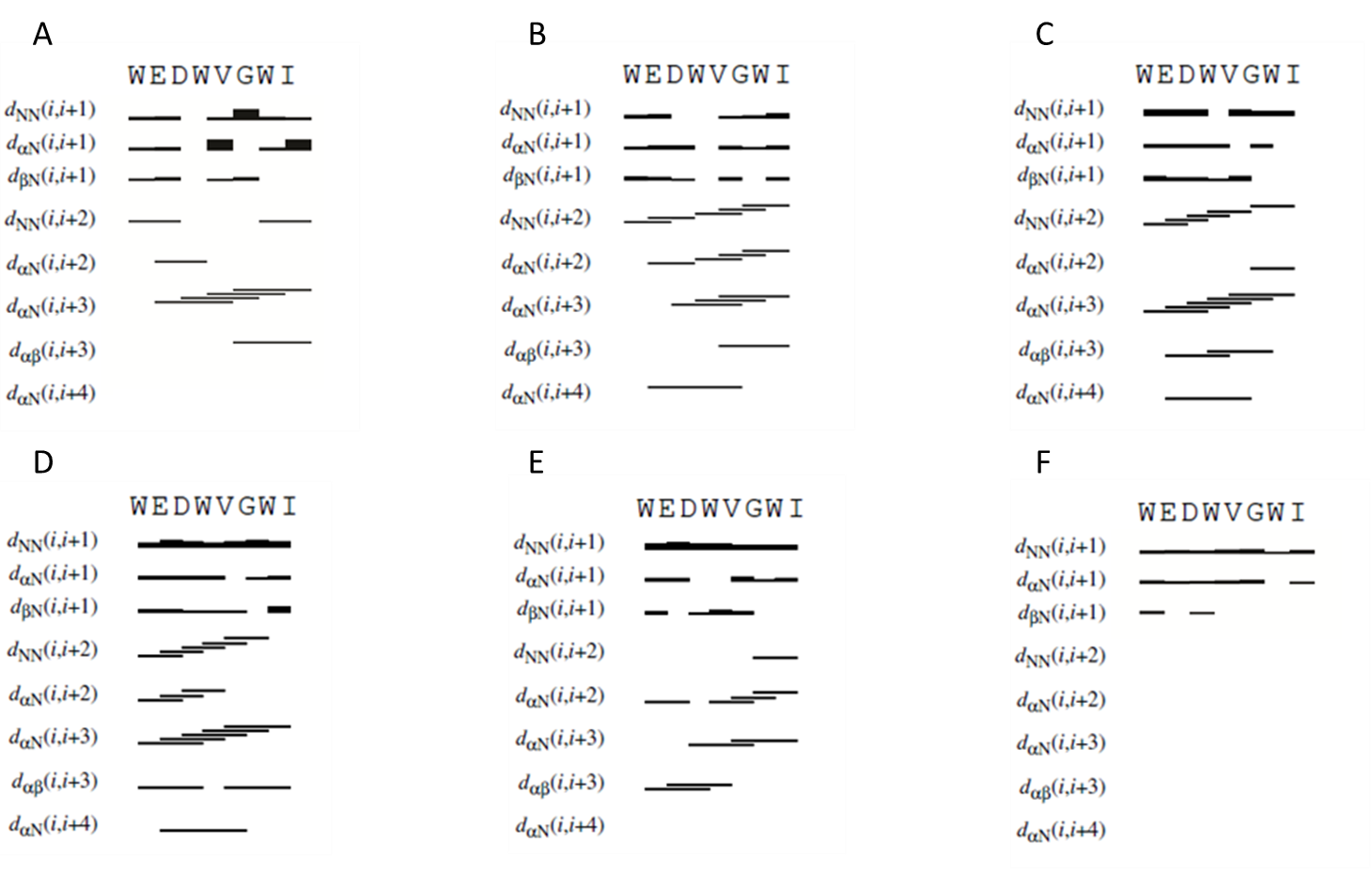


**Figure S3.** HN (A) and CHα (B) chemical shift change in in DPC/SDS 100:0, 90:10, 60:40, 40:60, 10:90 and 0:100 molar ratios. Data were obtained from a 600 MHz TOCSY experiments collected at 300 K.


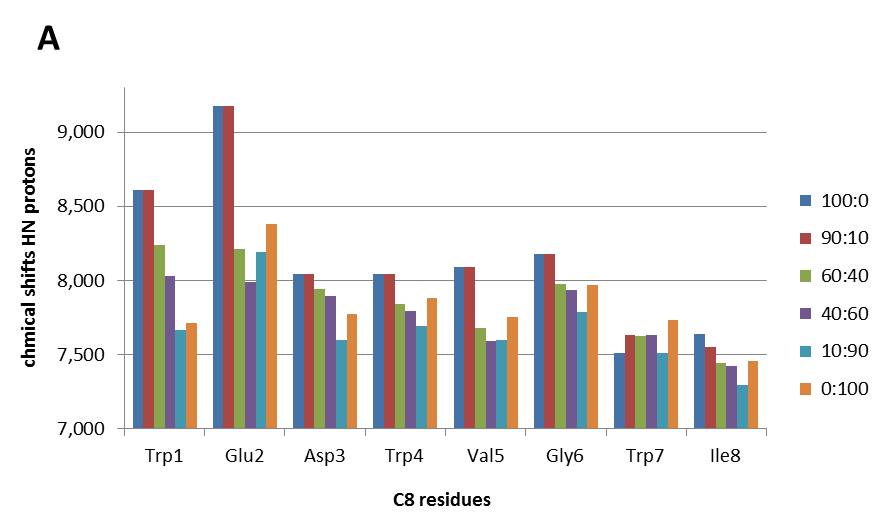


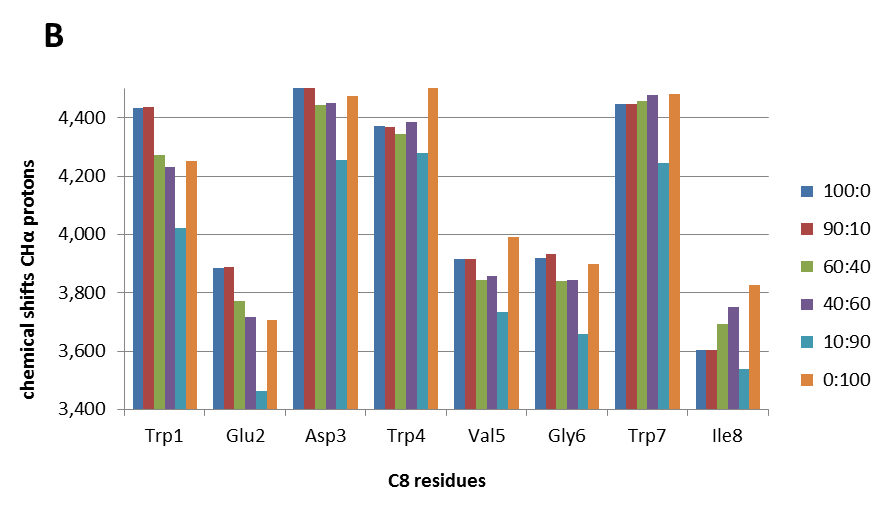


**Figure S4.** ^1^H backbone NH intensities of C8 in micelles composed of DPC/SDS 90:10 molar ratio recorded in the absence and the presence of 5-doxyl-stearic acid. Legends in the frames are relative to spin-label concentrations.

**Figure S5.** Superposition between the two most representative C8 binding conformations A and B derived from DOPC 100% simulation. The peptides structures are depicted as cartoon. The 6 Cα atoms on which the RMSD was calculated are colored in orange whereas the remaining two in grey.


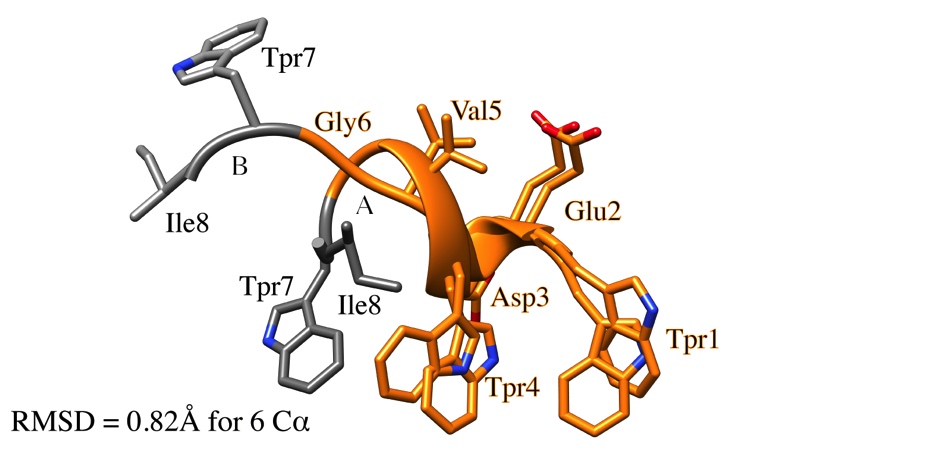


**Figure S6.** (A) The selected binding modes for the C8 peptide in DOPC 100% are reported with the membrane. The phospholipids are represented as a transparent surface. The aliphatic chains are colored in yellow, whereas the polar heads of DOPC are colored in light blue. The DOPC polar heads interacting with C8 are also reported in the grey stick. The electrostatic interactions are highlighted with dashed black lines. The distances between the carbon atoms of the carboxylic groups and the positively charged nitrogen atoms of the phosphatidylcholine groups are also reported (A) as in (B), but the represented C8 peptide binding mode was extracted from the DOPC/DOPG 90:10 (%/%) and the polar heads of DOPG are colored in light grey.


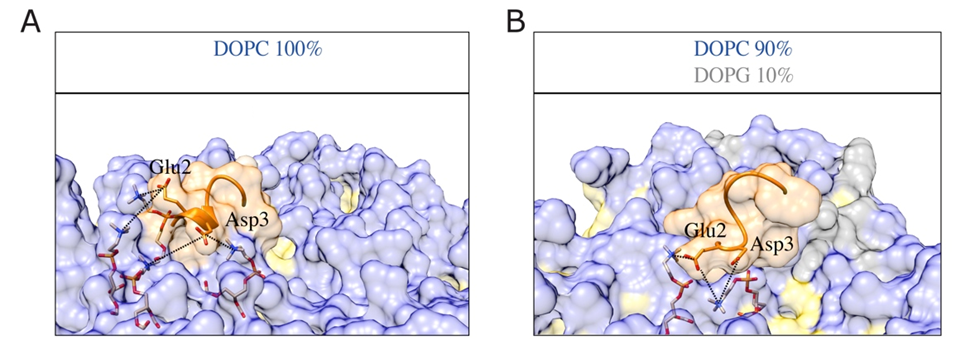


**Figure S7.** Interactions engaged by C8 with the membrane atoms in DOPC (A) and DOPC/DOPG 90/10 (%/%) (B). The interatomic contacts were first measured using a switching function and then the percentage of existence for each interaction was evaluated within the C8/membrane bound states present in cluster 1 in DOPC and cluster 1 in DOPC/DOPG 90/10 (%/%).


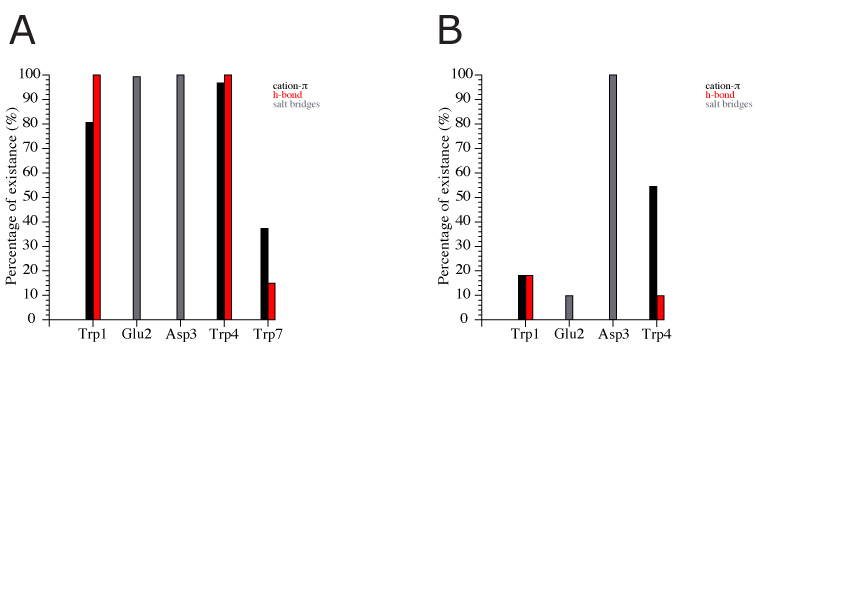


**Figure S8.** The umbrella PMF calculated for the translocation of the C8 peptide through pure DOPC bilayer The bars represent the histograms computed in the different simulation windows. Z = 0.0 Å corresponds to the centre of the bilayer. The colour code represents the different simulation times for which the PMF is calculated.

**
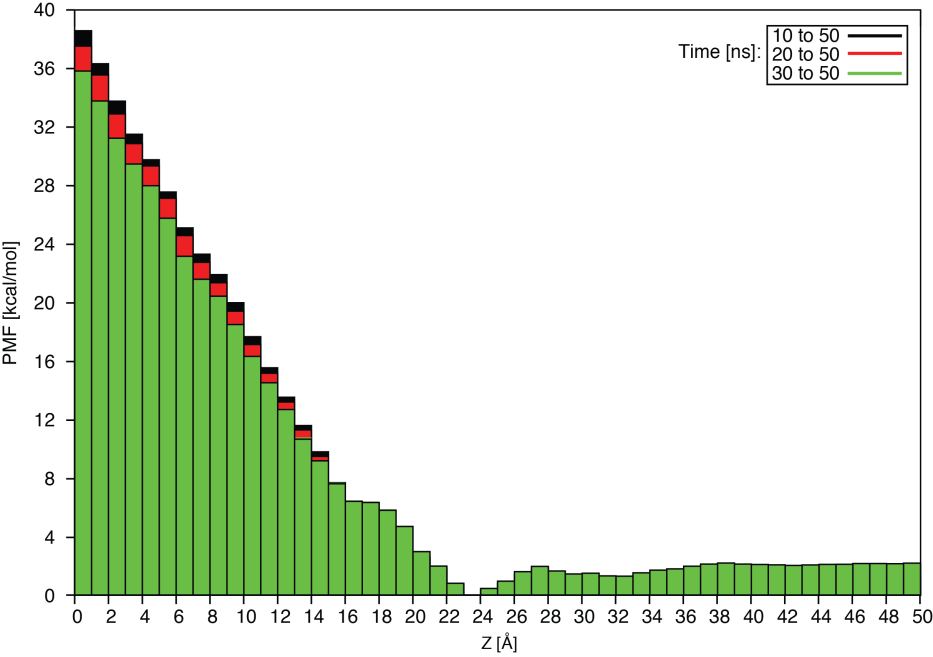
**

**References**

Scrima, M., Di Marino, S., Grimaldi, M., Campana, F., Vitiello, G., Piotto, S.P., et al. (2014). Structural features of the C8 antiviral peptide in a membrane-mimicking environment. *Biochim Biophys Acta* 1838(3)**,** 1010-1018. doi: 10.1016/j.bbamem.2013.12.010.
